# Small Extracellular Vesicles Secreted by Nigrostriatal Astrocytes Rescue Cell Death and Preserve Mitochondrial Function in Parkinson's Disease

**DOI:** 10.1002/adhm.202201203

**Published:** 2022-08-15

**Authors:** Loredana Leggio, Francesca L'Episcopo, Andrea Magrì, María José Ulloa‐Navas, Greta Paternò, Silvia Vivarelli, Carlos A. P. Bastos, Cataldo Tirolo, Nunzio Testa, Salvatore Caniglia, Pierpaolo Risiglione, Fabrizio Pappalardo, Alessandro Serra, Patricia García‐Tárraga, Nuno Faria, Jonathan J. Powell, Luca Peruzzotti‐Jametti, Stefano Pluchino, José Manuel García‐Verdugo, Angela Messina, Bianca Marchetti, Nunzio Iraci

**Affiliations:** ^1^ Department of Biomedical and Biotechnological Sciences University of Catania Catania 95123 Italy; ^2^ Oasi Research Institute‐IRCCS Troina 94018 Italy; ^3^ Department of Biological, Geological and Environmental Sciences University of Catania Catania 95125 Italy; ^4^ Laboratory of Compared Neurobiology University of Valencia‐CIBERNED Paterna 46980 Spain; ^5^ Department of Neuroscience Mayo Clinic Jacksonville FL 32257 USA; ^6^ Department of Veterinary Medicine University of Cambridge Cambridge CB3 0ES UK; ^7^ Luminex B.V. ‘s‐Hertogenbosch 5115 MV The Netherlands; ^8^ Department of Clinical Neurosciences University of Cambridge Cambridge CB2 0QQ UK

**Keywords:** astrocytes, exosomes, extracellular vesicles, high‐resolution respirometry, mitochondria, Parkinson's disease

## Abstract

Extracellular vesicles (EVs) are emerging as powerful players in cell‐to‐cell communication both in healthy and diseased brain. In Parkinson's disease (PD)—characterized by selective dopaminergic neuron death in ventral midbrain (VMB) and degeneration of their terminals in striatum (STR)—astrocytes exert dual harmful/protective functions, with mechanisms not fully elucidated. Here, this study shows that astrocytes from the VMB‐, STR‐, and VMB/STR‐depleted brains release a population of small EVs  in a region‐specific manner. Interestingly, VMB‐astrocytes secreted the highest rate of EVs, which is further exclusively increased in response to CCL3, a chemokine that promotes robust dopaminergic neuroprotection in different PD models. The neuroprotective potential of nigrostriatal astrocyte‐EVs is investigated in differentiated versus undifferentiated SH‐SY5Y cells exposed to oxidative stress and mitochondrial toxicity. EVs from both VMB‐ and STR‐astrocytes counteract H_2_O_2_‐induced caspase‐3 activation specifically in differentiated cells, with EVs from CCL3‐treated astrocytes showing a higher protective effect. High resolution respirometry further reveals that nigrostriatal astrocyte‐EVs rescue neuronal mitochondrial complex I function impaired by the neurotoxin MPP^+^. Notably, only EVs from VMB‐astrocyte fully restore ATP production, again specifically in differentiated SH‐SY5Y. These results highlight a regional diversity in the nigrostriatal system for the secretion and activities of astrocyte‐EVs, with neuroprotective implications for PD.

## Graphical abstract

Astrocytes from the main brain regions affected by Parkinson's disease (PD) secrete different levels of extracellular vesicles (EVs). CCL3‐EVs better protect neurons from apoptosis and only ventral midbrain‐derived EVs ameliorate ATP production in neurotoxin‐injured neurons. These data demonstrate the importance of the brain region for the neuroprotection mediated by astrocyte‐EVs, with implications for PD diagnosis and treatment.

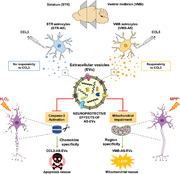

## 1 Introduction

Extracellular vesicles (EVs) are nanometric (30–1000 nm) lipid membranous structures released by virtually all cell types into the extracellular milieu, where they can be captured by adjacent or distal cells.^[^ ^1^, ^2^, ^3^, ^4^ ^]^ EVs is a general term used to describe a complex set of vesicles with distinct biogenesis and release mechanisms. Exosomes, microvesicles and apoptotic bodies partially overlap in terms of dimension and composition, making it difficult to identify specific EV subclasses.^[^ ^5^ ^]^ Based on size, EVs are referred to as medium‐large (>200 nm) or small (<200 nm, sEVs).^[^ ^5^, ^6^, ^7^ ^]^ The importance of EVs in mediating cell‐to‐cell communication resides in their ability to deliver different cargoes (i.e., nucleic acids, proteins, metabolites, lipids) to target cells, thus influencing the cellular fate.^[^ ^8^, ^9^, ^10^, ^11^, ^12^ ^]^ EVs have been identified in body fluids as potential new biomarkers for several diseases and are also exploited as advanced nanotherapeutics in regenerative medicine.^[^ ^13^, ^14^, ^15^, ^16^, ^17^, ^18^, ^19^, ^20^, ^21^, ^22^, ^23^, ^24^, ^25^ ^]^ Like their synthetic liposomal counterpart, EVs protect their payloads from the action of nucleases and proteases, allowing the delivery to distant sites.^[^ ^20^ ^]^ Also, EVs display innate properties, such as the ability to cross biological barriers (e.g., blood brain barrier, although the mechanisms are not fully elucidated),^[^ ^26^, ^27^ ^]^ and a potential low immunogenicity.^[^ ^28^, ^29^ ^]^ Moreover, EVs possess a fingerprint, inherited from their donor cells, that distinguishes vesicles derived from different cell types.^[^ ^20^, ^30^, ^31^, ^32^ ^]^ Importantly, the EV content: i) reflects the “status” of the donor cell; and ii) it can change in response to specific modifications in the microenvironment.^[^ ^33^ ^]^

EVs have been demonstrated to play several roles in physio‐pathological conditions.^[^ ^34^ ^]^ In the context of neurodegenerative diseases, including Parkinson's disease (PD), EVs were initially identified as vehicles of misfolded proteins,^[^ ^35^, ^36^, ^37^ ^]^ but in line with the dual role played by glial cells, EVs have been demonstrated to play also important neuroprotective functions.^[^ ^38^, ^39^ ^]^

In particular, astrocyte (AS) dysfunction is increasingly emerging as a critical feature of PD,^[^ ^40^, ^41^, ^42^, ^43^, ^44^, ^45^, ^46^, ^47^, ^48^, ^49^, ^50^, ^51^ ^]^ the second most common neurodegenerative disorder, with no available cure to stop or reverse its progression.^[^ ^52^ ^]^ PD is characterized by the selective and unrestrained death of dopaminergic (DAergic) cell bodies of the substantia nigra pars compacta (SNpc), residing in the ventral midbrain (VMB).^[^ ^52^, ^53^, ^54^ ^]^ As a consequence, in the striatum (STR), DAergic terminals slowly degenerate leading to the classical motor features of PD (i.e., bradykinesia, rest tremor, rigidity, and postural instability).^[^ ^52^, ^53^, ^54^, ^55^ ^]^ Along with the chronic, age‐dependent nigrostriatal degeneration, the abnormal accumulation of intraneuronal inclusions enriched in aggregated *α*‐synuclein (*α*‐syn), known as Lewy bodies (LBs) and Lewy neurites (LNs), as well as a massive astrogliosis, represent the major histopathologic hallmarks of the disease.^[^ ^55^, ^56^, ^57^, ^58^ ^]^ The causes and mechanisms of DAergic neuron death still remain elusive, albeit current evidence implicate a complex interplay between several genes and many environmental factors, especially ageing, inflammation and oxidative stress, all robustly impacting the astroglial cell compartment.^[^ ^40^, ^41^, ^44^, ^45^, ^46^, ^48^, ^59^, ^60^, ^61^, ^62^, ^63^ ^]^ Converging data point to mitochondrial dysfunction as the pivotal final pathway for PD neurodegeneration, closely related to the selective vulnerability of nigrostriatal neurons and to the specific properties of the astroglial microenvironment.^[^ ^63^, ^64^, ^65^, ^66^, ^67^, ^68^, ^69^, ^70^, ^71^, ^72^, ^73^, ^74^ ^]^ In fact, AS are active mediators of either beneficial or detrimental functions during neuronal degeneration, via the expression of a plethora of proinflammatory/anti‐inflammatory molecules and neurotoxic/neuroprotective mediators.^[^ ^41^, ^44^, ^45^, ^71^, ^72^, ^75^, ^76^ ^]^ The balance between these messengers, together with the bidirectional signaling with microglial cells, will determine the fate either toward a reparative process or a neuronal failure.

In this context, growing evidence highlights regional AS heterogeneity, at both molecular and functional levels, with important consequences for neuronal function and/or vulnerability.^[^ ^77^, ^78^, ^79^, ^80^, ^81^, ^82^, ^83^, ^84^, ^85^, ^86^, ^87^ ^]^ Of note, AS display unique features within the nigrostriatal system, since within the central nervous system (CNS), VMB and STR are highly sensitive to oxidative stress, environmental toxins, inflammatory challenges, and ageing.^[^ ^40^, ^44^, ^85^, ^87^, ^88^, ^89^, ^90^ ^]^ Also, AS exhibit regional heterogeneity in response to cytokines and chemokines during neuroinflammation and neurodegeneration/neuroprotection, with increasing evidence pointing at chemokines as major mediators of glia‐neuron crosstalk.^[^ ^91^, ^92^, ^93^ ^]^

Accordingly, within the VMB, AS exert potent neuroprotective effects toward the vulnerable SNpc‐DAergic neurons (reviewed in Ref. [73]). In particular, reactive VMB‐AS were identified as main actors linking neuroinflammation to DAergic neuroprotection and repair in the 1‐methyl, 4‐phenyl, 1,2,3,6 tetrahydropyridine (MPTP) mouse model of basal ganglia injury.^[^ ^94^ ^]^ In this context, a wide gene expression analysis identified a major upregulation of certain chemokines, in particular the CC chemokine ligand 3 (CCL3), shown to play important roles for DAergic neurogenesis, survival, and immunomodulation.^[^ ^94^, ^95^, ^96^ ^]^ Notably, in vitro studies revealed CCL3‐activated AS‐neuron crosstalk as a critical element promoting both neuroprotection and neurogenesis of adult neural stem cells (NSCs).^[^ ^44^, ^73^, ^94^ ^]^ However, the molecular details of this complex intercellular signaling are still a matter of intense debate. Here, we scrutinized the secretion of AS‐derived extracellular vesicles (AS‐EVs) from the VMB versus STR brain regions, in both basal and CCL3‐activated conditions, as a likely way of communication with injured DAergic neurons. For the first time, our study demonstrates that AS‐EVs in the nigrostriatal system propagate specific neuroprotective intercellular signaling, targeting neural oxidative damage and mitochondrial dysfunction, with region‐dependent functional implications. This potential may be exploited to enhance the neuroprotection of DAergic neurons in PD.

## 2 Results

### 2.1 Astrocytes from the Nigrostriatal System Secrete Small EVs in a Region‐Specific Manner

To assess potential differences in AS‐EVs between the two principal brain regions affected in PD, primary AS cultures were established from the VMB and STR (Figure S1, Supporting Information).^[^ ^94^ ^]^ Also, AS were grown from brains depleted of these two regions (hereafter called ΔVS‐AS), as control cells external to the nigrostriatal system (Figure S1, Supporting Information). AS were characterized under both basal and CCL3‐treated (24 h) conditions, to test whether the latter confers additional protective effects to AS‐EVs.^[^ ^94^ ^]^ All primary cultures produced high purity AS (≥98% of GFAP^+^ cells), without any differences in terms of proliferation rate upon CCL3 treatment (Figure S1A–D, Supporting Information). Moreover, in order to evaluate the health of AS at the time of EV isolation, the cell viability/death levels were investigated, and no significant differences were found between experimental groups, demonstrating that these factors are not in play to influence the production rate of AS‐EVs from different brain regions (Figure S1E, Supporting Information).

**Figure 1:**
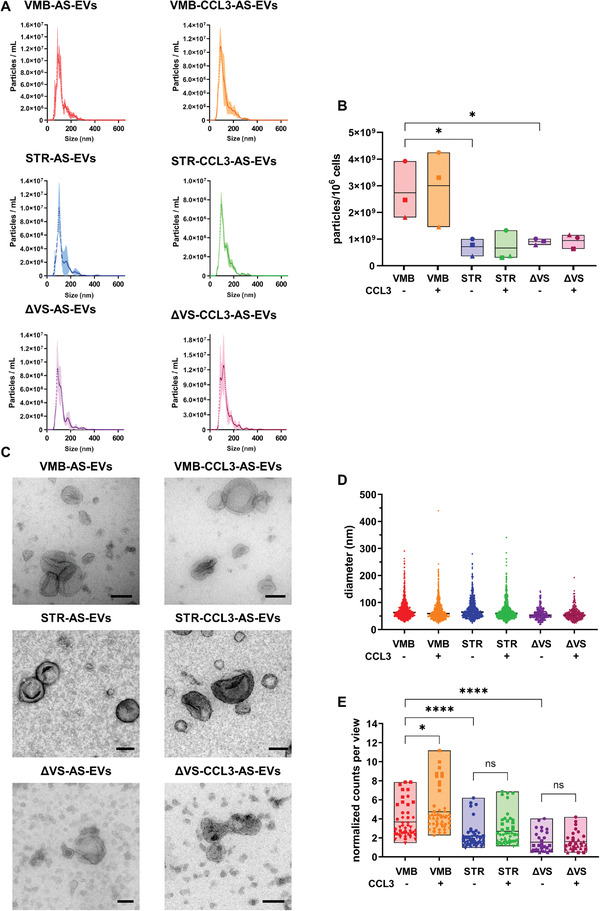
Brain region influences the rate of secretion of AS‐sEVs and responsiveness to CCL3 treatment. A) NTA analysis for size distribution displays a peak ≈100 nm. Error bars represent SD from *n* = 3 independent replicates. B) EV concentration, determined by NTA, was normalized over the number of cells. The mean of particles/10^6^ cells shows that astrocytes from VMB region secrete more EVs than STR and ΔVS regions. Data are presented as floating bars with line at mean from *n* = 3 independent replicates, indicated with different symbols. One‐way ANOVA with Tukey's multiple comparison ∗*p* < 0.05 (VMB‐AS‐EVs versus STR‐AS‐EVs; VMB‐AS‐EVs versus ΔVS‐AS‐EVs). C) TEM ultrastructural analysis reveals the presence of sEVs secreted by AS in every condition. Scale bars: 100 nm. D) In all AS‐EV samples the average diameter is ≈60/70 nm. Raw data (diameter values) are presented as scatter dot plots with line at median ± SD from *n* = 5 (for VMB‐ and STR‐AS‐EVs) and *n* = 3 (for ΔVS‐AS‐EVs) independent experiments. E) Quantitative analysis from TEM showed that astrocytes from VMB secrete more EVs than STR and ΔVS regions; the treatment with CCL3 stimulates VMB‐AS to release more EVs. Data are normalized considering the number of starting cells, the resuspension volume after ultracentrifugation, the volume used in the microscope grid, and the area (µm^2^) of each field in the grid. Data are presented as floating bars with line at mean plus individual data points based on 50 images over 5 independent replicates (for VMB‐ and STR‐AS‐EVs) and on 30 images over 3 independent replicates (for ΔVS‐AS‐EVs), indicated with different symbols. One‐way ANOVA with Tukey's multiple comparison: in (B) ∗*p* < 0.05 (VMB‐AS‐EVs versus STR‐AS‐EVs and versus ΔVS‐AS‐EVs; in (E) ∗*p* < 0.05 (VMB‐AS‐EVs versus VMB‐CCL3‐AS‐EVs), ∗∗∗∗*p* < 0.0001 (VMB‐AS‐EVs versus STR‐AS‐EVs and ΔVS‐AS‐EVs), ns: not significant.

Next, EVs were isolated from AS supernatants by differential centrifugation^[^ ^97^, ^98^ ^]^ and analyzed using a combination of different techniques, in order to evaluate dimensions, secretion rates, and specific markers. As a first line of characterization, we performed nanoparticle tracking analysis (NTA) on all EV samples. The data displayed an enriched population of vesicles with a peak size ≈100 nm, in the range of sEVs (**Figure** **1**A). Interestingly, we observed that brain region of origin impacts on the EV secretion rate of astrocytes, since VMB‐AS released 2‐ to 4‐fold more vesicles per million cells compared to STR and ΔVS, with an increased trend following CCL3 treatment (Figure 1B).

To further investigate this finding, we performed transmission electron microscopy (TEM) analysis on the same EV samples. The images show the presence of sEVs with the cup shapes, a typical result of the ultracentrifugation process (Figure 1C), with an average diameter between 60 and 70 nm (Figure 1D and Table S1, Supporting Information). Again, we found that VMB‐AS released more vesicles than astrocytes from STR and ΔVS (Figure 1E), corroborating the NTA results. Interestingly, we observed that the treatment with CCL3 stimulated VMB‐AS to secrete more EVs (Figure 1E), while the other two regions did not show any significant change in the secretion rate following the CCL3 treatment (Figure 1E).

To study the differential responses to CCL3 between the three types of astrocytes, we evaluated the expression of the main chemokine receptors, Ccr1 and Ccr5,^[^ ^99^, ^100^ ^]^ by quantitative real‐time PCR (qPCR). Ccr1 and Ccr5 levels were comparable between VMB and STR, and unchanged after treatment with CCL3 (Figure S2A, Supporting Information). In contrast, Ccr1 and Ccr5 expression in ΔVS‐AS samples was ≈100 times lower than in VMB‐ and STR‐AS, with no change upon chemokine treatment (Figure S2A, Supporting Information). These data suggest that only the main regions involved in PD, namely VMB and STR, have the potential to respond to treatment with CCL3, corroborating previous findings.^[^ ^40^, ^94^, ^101^, ^102^, ^103^ ^]^ (Figure S2A, Supporting Information). Such a limited capacity of ΔVS‐AS to respond to our stimulation protocol, prompted us to focus on the nigrostriatal system to further explore whether CCL3 elicits any cellular response in VMB‐ and STR‐AS. Histological analysis performed in 1.5 µm sections showed that CCL3 induces STR‐AS to shift to a more pronounced irregular membrane morphology compared to VMB‐AS (Figure S2B, Supporting Information). Moreover, scanning electron microscopy (SEM) analyses evidenced the remarkable presence of connectivity/secretory structures (e.g., microvilli‐like processes) at the cellular surface of both VMB‐ and STR‐AS (Figure S2C, Supporting Information). Again, STR‐CCL3‐AS exhibit a higher number of these membrane protrusions, suggesting that both brain regions are able respond to CCL3, but with a different outcome (Figure S2C, Supporting Information). Interestingly, this responsiveness involves changes in membrane dynamics, in line with published evidences regarding CCL3.^[^ ^104^, ^105^ ^]^

Overall, these data demonstrate that AS‐EV secretion characteristics are defined by their brain area origins.

### 2.2 Both VMB‐ and STR‐AS‐Derived Vesicles Are Enriched in sEV Markers

**Figure 2:**
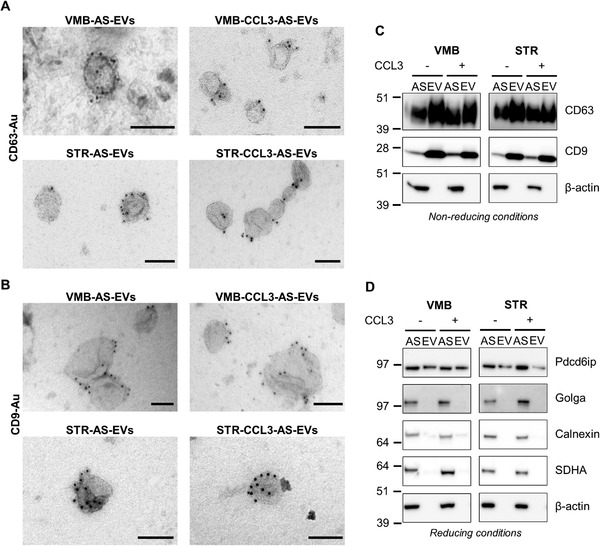
AS secrete vesicles enriched in sEV markers. A,B) IG‐TEM on EV samples with *α*‐CD63 (A) and *α*‐CD9 (B). Scale bars: 100 nm. C,D) WB analyses on EV lysates and corresponding AS donor cells. WBs for *α*‐CD63/CD9 ((C), in non‐reducing conditions) and for Pdcd6ip ((D), in reducing conditions) show an enrichment in the EV samples versus donor AS. On the contrary, the cellular markers (i.e., Golga, Calnexin, SDHA, and Actin) are mostly enriched in AS (D). All panels are representative of *n* = 3 independent experiments showing the same trend.

Next, we investigated the protein profiles of AS‐EVs from the nigrostriatal system. We applied immunogold‐labelling TEM (IG‐TEM) for the tetraspanins CD63 and CD9, as sEV markers. The images in **Figure** **2**A,B revealed the presence of both proteins, visualized as well‐defined 6 nm gold nanoparticles at the EV surface. In order to extend these results to other sEV markers, and exclude contamination from other cellular components, we used western blotting (WB) (Figure 2C,D). In line with the immunogold‐labelling TEM (IG‐TEM) data, we found a specific enrichment of the tetraspanins CD63/CD9 and Pdcd6ip (Alix) in all EV samples compared to donor AS. In contrast, the cellular markers Golga2 (for Golgi), calnexin (for endoplasmic reticulum), SDHA (for mitochondria) and actin (for cytoplasm), were mainly retained in the cells (Figure 2C,D). These results confirm that our vesicular preparations are enriched in sEVs.

### 2.3 Both VMB‐ and STR‐AS‐Derived Vesicles Are Internalized by SH‐SY5Y Cells

**Figure 3:**
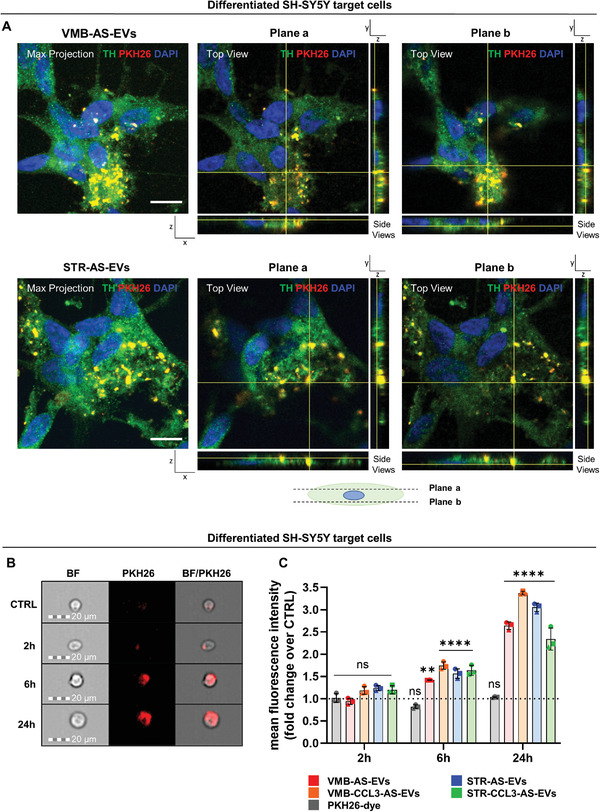
PKH26‐labelled AS‐EVs are internalized by differentiated, TH^+^ SH‐SY5Y neuronal cells. A) Max projection and orthogonal views of representative fields show the uptake of both VMB‐AS‐ and STR‐AS‐PKH26‐labelled EVs by differentiated SH‐SY5Y. Each max projection is composed of a stack of 15 individual *z* planes, acquired every 0.4 µm along the *z* axis. Scale bar 10 µm. Plane *a* and Plane *b* orthogonal views represent, respectively, two selected planes located above and below the cellular nuclei (along the *z* axis), as represented by the cellular schematic. In all panels PKH26 is in red, TH in green, whereas nuclear DAPI counterstain is in blue. Confocal images show that PKH26 labelled EVs are present within the cellular bodies of SH‐SY5Y target cells upon 6 h of incubation. B) Representative images from IFC of differentiated SH‐SY5Y treated with PKH26‐AS‐EVs for 2, 6, and 24 h. Magnification 20×, scale bar 20 µm. C) IFC analysis of differentiated SH‐SY5Y cells treated with PKH26‐AS‐EVs and PKH26‐dye‐only at different time points. Data are expressed as fold change of the mean fluorescence intensity ± SD over CTRL set to 1 for comparison (dotted line), from *n* = 3 independent experiments, indicated with different symbols. One‐way ANOVA with Tukey's multiple comparison versus CTRL. ∗∗*p* < 0.01, ∗∗∗∗*p* < 0.0001, ns: not significant.

Before examining the effects of the sEVs on target neurons, we evaluated their internalization using the human neuroblastoma SH‐SY5Y cells differentiated with retinoic acid (RA), as a model of tyrosine hydroxylase positive (TH^+^) neuronal target cells.^[^ ^106^, ^107^, ^108^ ^]^ To follow AS‐EVs, we used two different labeling approaches: i) both VMB‐ and STR‐AS were treated with the PKH26 membrane dye, followed by ultracentrifugation to isolate labelled EVs (**Figure** **3**A and Figure S3A,B, Supporting Information); and ii) PKH26 were applied directly to AS‐EVs after ultracentrifugation (Figure S3C, Supporting Information). Both approaches led to PKH26‐labelled AS‐EVs, which were then administered to target cells at a ratio of 5:1 (EVs derived from five astrocytes used to treat one target cells), in line with the local brain tissue architecture.^[^ ^109^, ^110^ ^]^ First, the capacity of differentiated SH‐SY5Y cells to internalize AS‐EVs was evaluated by confocal microscopy. As shown by the orthogonal view analyses reported in Figure 3A and Figure S3A, Supporting Information, PKH26‐labelled AS‐EVs were efficiently taken up by cells and partially co‐localized with TH, which has a high affinity for phospholipid membranes.^[^ ^111^ ^]^ A volumetric 3D reconstruction of the intracellular distribution of AS‐EVs confirms the effective enrichment of vesicles within the cytoplasmic compartment (Figure S3B and Movies S1 and S2, Supporting Information). Moreover, the bright field/IF combined view suggests that AS‐EVs are distributed in the whole cytoplasm, including neurite protrusions (Figure S3C, Supporting Information). Thus, AS‐EVs can be efficiently transferred to neuronal target cells.

Next, in order to quantify the internalization of the different vesicle samples by target cells, PKH26‐labelled AS‐EVs were administered to differentiated SH‐SY5Y followed by imaging flow cytometry (IFC),^[^ ^112^ ^]^ and their fluorescence intensity was measured after 2, 6 and 24 h (Figure 3B). Fluorescence increased in time dependent manner: i) at 2 h there was no significant difference between untreated (CTRL) and treated cells; ii) at 6 h fluorescence intensity increased significantly by 1.4‐ to 1.7‐fold versus CTRL; and iii) at 24 h PKH26 intensity further increased (Figure 3C), suggesting that AS‐EVs continued to enter target neurons (Figure 3C), in line with previous reports.^[^ ^113^ ^]^

Although it is not possible to exclude that PKH26 can label certain contaminant proteins in EV preparations,^[^ ^114^, ^115^ ^]^ it is unlikely that dye self‐aggregation interfered with the analysis of vesicle uptake, as dye‐only samples failed to label SH‐SY5Y cells at all time points and with both techniques (i.e., IFC and IF) (Figure 3C and Figure S3C, Supporting Information).

Finally, in order to evaluate whether the differentiation process may influence the uptake of AS‐EVs, we performed the same IFC analysis using undifferentiated SH‐SY5Y target cells. As shown in Figure S3D, Supporting Information, a similar uptake time‐course was observed for these cells (fluorescence intensity significantly rising by 1.4‐ to 1.8‐fold after 6 h, with the uptake at 24 h higher than at 6 h). Thus, AS‐EVs are internalized to a similar extent by SH‐SY5Y cells, regardless of the regional identity of donor astrocytes, or the differentiation state of target neurons.

### 2.4 EVs from CCL3‐Activated Astrocytes Prevent H_2_O_2_‐Induced Caspase‐3 Activation in Differentiated SH‐SY5Y Neurons

To evaluate the effects of AS‐EVs on target differentiated SH‐SY5Y cells under oxidative stress/neurodegenerative conditions, we used the hydrogen peroxide (H_2_O_2_),^[^ ^116^ ^]^ a general source of ROS, and the neurotoxin MPP^+^, both used as well‐established in vitro models of PD.^[^ ^117^, ^118^, ^119^ ^]^ Based on preliminary time‐course and dose‐response experiments, we applied 35 µm H_2_O_2_ or 1 mm MPP^+^ for 24 h, treatments which consistently reduced cell viability by ≈40% and ≈10%, respectively—without inducing an acute and massive cell death (Figure S4A,B, Supporting Information).^[^ ^120^, ^121^, ^122^ ^]^ Considering the internalization data, target cells were incubated with a 5:1 ratio of AS‐EVs 6 h prior to the challenge with H_2_O_2_ or MPP^+^, to allow a significant uptake of vesicles.

**Figure 4:**
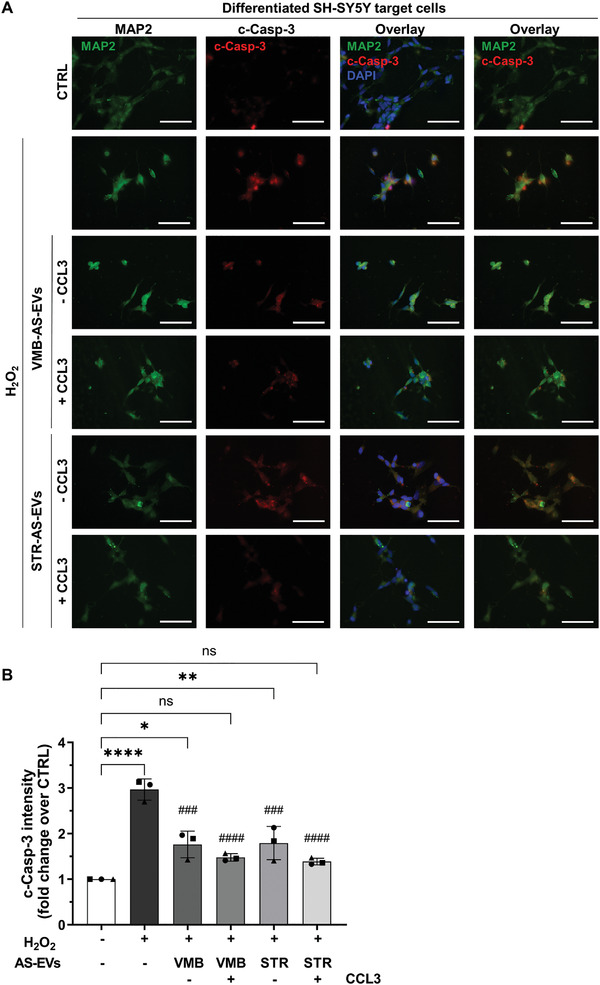
AS‐EVs significantly reduce apoptosis in differentiated SH‐SY5Y neurons challenged with H_2_O_2_. A) IF staining for MAP2 (in green), c‐Casp‐3 (in red), and DAPI (in blue), on differentiated SH‐SY5Y exposed to AS‐EVs and treated with 35 µm H_2_O_2_. Scale bars: 50 µm. B) Quantification of the c‐Casp‐3 staining in (A). The fluorescent intensities were normalized over the cell number. Data are expressed as mean ± SD over CTRL set to 1 for comparison, from *n* = 3 independent replicates, indicated with different symbols. One‐way ANOVA with Tukey's multiple comparison ∗*p* < 0.05, ∗∗*p* < 0.01, ∗∗∗∗*p* < 0.0001 versus CTRL, ns: not significant; ###*p* < 0.001, ####*p* < 0.0001 versus H_2_O_2_.

First, the extent of apoptosis was measured in differentiated SH‐SY5Y cells using cleaved caspase‐3 (c‐Casp‐3), whose levels were evaluated 24 h after H_2_O_2_ treatment via IF (**Figure** **4**A). Analysis of fluorescence intensity revealed that H_2_O_2_ induced a 3‐fold increase of c‐Casp‐3 compared to untreated cells (CTRL, Figure 4B). The presence of both VMB‐ and STR‐AS‐EVs significantly reduced apoptosis levels, with the EVs from CCL3‐treated AS showing a full rescue of the c‐Casp‐3 levels induced by H_2_O_2_ (Figure 4B). As a control, we applied CCL3 directly to H_2_O_2_‐injured SH‐SY5Y cells, without any rescue of cell viability (Figure S4C,D, Supporting Information). The same negative result was obtained by treating differentiated SH‐SY5Y cells with contaminant EVs (cont‐EVs) isolated from the complete medium only (i.e., medium that was not in contact with cells) (Figure S4E,F, Supporting Information).

To understand if the neuroprotective effect was specific for H_2_O_2_‐injured differentiated SH‐SY5Y cells, we measured caspase activity in undifferentiated SH‐SY5Y cells treated with the AS‐EVs, by using the same protocols (Figure S4G, Supporting Information). While H_2_O_2_ induced a 2.5‐fold increase of caspase activity, pre‐exposure to AS‐EVs did not reduce apoptosis induced by H_2_O_2_ (Figure S4G, Supporting Information). Together, these results further identify AS‐EVs as specific and effective mediators that deliver protective cargoes to H_2_O_2_‐injured differentiated SH‐SY5Y cells. The data also support the usefulness of CCL3‐activated astrocytes as neuroprotective agents in nigrostriatal degeneration.^[^ ^94^ ^]^

### 2.5 Both VMB‐ and STR‐AS‐Derived EVs Preserve the Activity of Mitochondrial Complex I in Differentiated SH‐SY5Y Neurons Injured by the Neurotoxin MPP^+^

**Figure 5:**
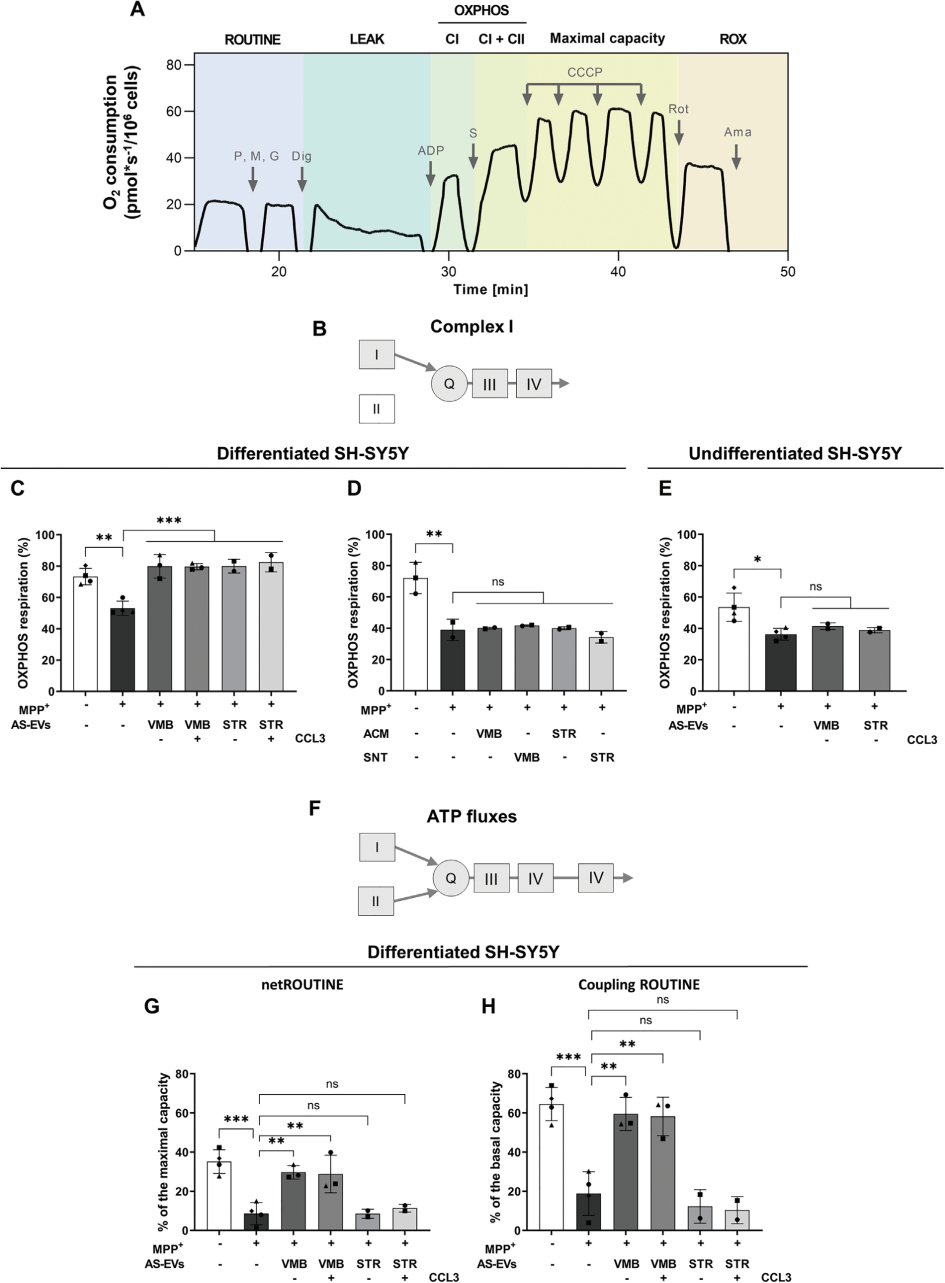
AS‐EVs recover mitochondrial functions in differentiated SH‐SY5Y neurons challenged with MPP^+^. A) Representative oxygraphic trace in untreated differentiated SH‐SY5Y (control) cells alongside the specific protocol used. First, in intact cells, the physiological O_2_ consumption, corresponding to ROUTINE state, was measured. Second, adenylates were forced to leave the cells by a mild plasma membrane permeabilization in order to analyze the LEAK state. Third, the contribution of CI to the OXPHOS respiration was assayed in the presence of the previous addition of the appropriate substrates (pyruvate, malate, glutamate) and a saturating ADP concentration. Then, addition of succinate allowed the activation of CII (CI + CII) and the achievement of total OXPHOS respiration. Fourth, the maximal capacity of ETS was obtained after CCCP titration. Fifth, the ROX was acquired after inhibition of ETS complexes with rotenone and antimycin A. P, pyruvate; M, malate; G, glutamate; Dig, digitonin; S, succinate; Rot, rotenone; Ama, antimycin A. B) Schematic representation of complex I activity measurement through mitochondrial ETS complexes. C) The effects of MPP^+^ and EVs were tested in the same experimental conditions. The toxin reduces CI activity (CI‐linked OXPHOS) of ≈30% compared to CTRL while AS‐EVs fully recover CI functionality of MPP^+^‐treated SH‐SY5Y cells. The OXPHOS respiration linked to CI was expressed as flux control ratio using the total OXPHOS respiration as reference state. One‐way ANOVA with Tukey's multiple comparison ∗∗*p* < 0.01 (CTRL versus MPP^+^), and ∗∗∗*p* < 0.001 (MPP^+^ versus MPP^+^ + VMB‐AS‐EVs ± CCL3 and versus MPP^+^ + STR‐AS‐EVs ± CCL3). D) The effects of ACM/SNT were tested as before. No significant differences were observed in MPP^+^‐injured cells treated with ACM or SNT samples. The OXPHOS respiration linked to CI was expressed as flux control ratio using the total OXPHOS respiration as reference state. One‐way ANOVA with Tukey's multiple comparison ∗∗*p* < 0.01 (CTRL versus MPP^+^), ns: not significant. E) The effects of AS‐EVs were tested in undifferentiated SH‐SY5Y cells. The toxin treatment significantly reduces CI activity versus CTRL, while no significant effect was observed in presence of AS‐EV samples. The OXPHOS respiration linked to CI was expressed as flux control ratio using the total OXPHOS respiration as reference state. One‐way ANOVA with Tukey's multiple comparison ∗*p* < 0.05 (CTRL versus MPP^+^), ns: not significant. In panels (C–E) data are expressed as the ratio between OXPHOS driven by CI and total OXPHOS (driven by CI + CII) ± SD. F) Schematic representation of mitochondrial ETS and ATP synthase complexes. G) MPP^+^ reduces O_2_ flux devoted to ATP production compared to CTRL in ROUTINE. Only VMB‐AS‐EVs promote a significant recovery of the flux in differentiated SH‐SY5Y cells. Net ROUTINE was expressed as flux control ratio using the maximal capacity as reference state. Data are expressed as percentage of the maximal ETS capacity ± SD. One‐way ANOVA with Tukey's multiple comparison, ∗∗*p* < 0.01 (MPP^+^ versus MPP^+^ + VMB‐AS‐EVs ± CCL3), ∗∗∗*p* < 0.001 (CTRL versus MPP^+^), ns: not significant. H) Coupling efficiency in basal state (ROUTINE) of differentiated SH‐SY5Y cells in the same experimental conditions. According to the ATP production data, only VMB‐AS‐EVs were able to significantly increase the rate of coupling between the oxidative phosphorylation and ATP production. Data are expressed as percentage of each specific state. Coupling efficiency was expressed as flux control ratio using the basal respiration (ROUTINE) as reference state. Data are expressed as means ± SD. One‐way ANOVA with Tukey's multiple comparison, ∗∗*p* < 0.01 (MPP^+^ versus MPP^+^ + VMB‐AS‐EVs ± CCL3), ***p < 0.001 (CTRL versus MPP^+^), ns: not significant.

Next, we extended the study of the neuroprotective potential of AS‐EVs to the same target cells when exposed to the neurotoxin MPP^+^. MPP^+^ affects DAergic neurons through the induction of a parkinsonian‐like phenotype mainly by inhibiting the activity of the mitochondrial NADH‐ubiquinone oxidoreductase (complex I, CI) of the electron transport chain.^[^ ^117^, ^123^ ^]^ Furthermore, as we recently demonstrated, the toxin compromises the overall integrity of the inner mitochondrial membrane (IMM), affecting ATP production via a mechanism which is independent from CI inhibition.^[^ ^106^ ^]^ As mentioned above, we selected the dose of 1 mm MPP^+^, which causes only a small (≈10%) reduction of cell viability after a 24 h incubation,^[^ ^106^ ^]^ thus excluding non‐specific mitochondrial deficits caused by a massive cell death process (Figure S4B, Supporting Information). EVs were applied on target cells (ratio 5:1) 6 h before the MPP^+^ challenge, and mitochondrial functionality was analyzed by high‐resolution respirometry (HRR) 24 h later.^[^ ^124^, ^125^ ^]^ We obtained the complete respiratory profile (i.e., the cellular O_2_ consumption upon addition of substrates/inhibitors) (see **Figure** **5**A for a representative trace of CTRL cells alongside a detailed protocol), and the main respiratory states of the different experimental groups were analyzed. This was achieved by two distinct steps: i) the mild permeabilization of plasma membranes, which allows the exit of substrates and thus the complete inhibition of OXPHOS respiration; and ii) the stimulation of CI activity with pyruvate, malate, glutamate, and ADP at saturating concentration (Figure 5A). This set‐up allows the electrons to flow from CI—but not from CII—to CIII, through the Q junction (Figure 5A,B). Only the subsequent addition of succinate enables CII to participate to the total OXPHOS respiration (Figure 5A,F).

MPP^+^ treatment did not change O_2_ consumption in both intact (ROUTINE state) or permeabilized and fully stimulated cells (OXPHOS state) (Figure S5A,B, Supporting Information). Contrariwise, it specifically affected the contribution of CI to the OXPHOS respiration (Figure 5B–E), as expected.

As shown in Figure 5C, in CTRL cells CI accounted for ≈73% of the overall OXPHOS, while MPP^+^ reduced its activity up to a value of ≈53%. In this context, all AS‐EV samples promoted a significant increase of CI activity in MPP^+^‐injured cells, up to full rescue (Figure 5C). Considering that the ultracentrifugation process may damage EVs with the consequent leakage of their content,^[^ ^126^ ^]^ control experiments were performed using both the astrocyte conditioned media (ACM, still containing EVs, but ≈50 times more diluted), or the same media depleted of EVs, obtained after ultracentrifugation (supernatant, SNT) (Figure 5D). In both cases, no significant rescue of CI activity was observed in MPP^+^‐injured cells, thereby confirming that only purified intact EVs are able to revert the MPP^+^‐induced complex I inhibition.

Because the EV uptake was similar in both differentiated and undifferentiated SH‐SY5Y, we next repeated HRR measurements in undifferentiated SH‐SY5Y exposed to 1 mm MPP^+^.

Although undifferentiated SH‐SY5Y showed slight differences in the overall respirometry profile in terms of absolute values, MPP^+^ treatment did not affect significantly ROUTINE or OXPHOS (Figure S5C,D, Supporting Information), but only CI activity, as for the differentiated cells. In particular, O_2_ consumption linked to CI ranged from ≈54% of CTRL cells to ≈36% of MPP^+^‐injured cells, but the treatment with AS‐EVs was ineffective in improving CI activity (Figure 5E).

Together, these data indicate the ability of AS‐EVs from both VMB and STR to efficiently preserve CI activity, specifically in differentiated SH‐SY5Y cells, at a concentration far below MPP^+^‐induced massive cytotoxicity.

### 2.6 Only EVs Secreted by VMB‐AS Ameliorate ATP Production in Differentiated, MPP^+^‐Injured SH‐SY5Y Neurons

Given the potential of all AS‐EV samples to protect CI activity from MPP^+^, we further assessed whether they positively impact on other critical features of MPP^+^‐induced mitochondrial dysfunction. As already shown in Figure S5A,B, Supporting Information, the neurotoxin does not affect the total O_2_ consumption recorded in the presence of endogenous substrates in intact cells, neither the one observed in the presence of externally added substrates in permeabilized cells. On the other hand, the neurotoxin treatment dramatically reduces the ATP‐related fluxes, also known as net fluxes.^[^ ^106^ ^]^ With this perspective, HRR was used to analyze the effect of AS‐EVs on the O_2_ flux exclusively devoted to ATP production in intact cells. As displayed in Figure 5G, MPP^+^ drastically reduced the net flux up to −75% in comparison to CTRL cells. Remarkably, treatment with EVs from VMB‐AS—but not from STR‐AS—significantly ameliorated the reduction in ADP phosphorylation of MPP^+^‐injured differentiated SH‐SY5Y cells, with no significant differences between basal and CCL3 conditions (Figure 5G). In line with these data, the degree of coupling between oxidative phosphorylation and electron flows (the coupling efficiency) was fully restored alongside with the increased ATP‐related flows with VMB‐AS‐EVs only (Figure 5H). Again, no significant variation in the net flux or coupling efficiency was observed neither upon treatment with ACM/SNT (Figure S5E,F, Supporting Information), nor when AS‐EVs were given to undifferentiated SH‐SY5Y cells (Figure S5G,H, Supporting Information).

Overall, these data indicate a regional specificity of VMB‐ versus STR‐AS‐EVs in their ability to rescue the mitochondrial functional capacity of differentiated SH‐SY5Y cells under MPP^+^ injury.

## 3 Discussion

Reactive AS are increasingly emerging as key players in the Parkinsonian brain, exerting both “beneficial” and “detrimental” effects.^[^ ^40^, ^41^, ^42^, ^44^, ^45^, ^46^, ^47^, ^48^, ^49^, ^50^, ^73^, ^89^, ^94^, ^127^, ^128^ ^]^ Especially, the heterogeneous nature of AS has been emphasized, showing regional AS differential responses to both genetic and environmental factors, including ageing, inflammatory or neurotoxin exposures, all crucial conditions of vulnerability for PD.^[^ ^40^, ^41^, ^44^, ^45^, ^73^, ^81^, ^88^, ^129^ ^]^ Yet, the modality of the intricate AS‐neuron crosstalk still remains undefined. Among the multiple modes of intercellular communication, the secretion of EVs has emerged as a powerful tool for the exchange of information.^[^ ^39^, ^130^, ^131^ ^]^ EVs are released by most cell types of the brain and they have also been identified in body fluids as potential new biomarkers for PD and other neurodegenerative diseases (NDs).^[^ ^13^, ^14^, ^18^, ^19^, ^20^, ^21^, ^132^ ^]^ Also, they are exploited as advanced nanotherapeutics in regenerative medicine.^[^ ^13^, ^15^, ^20^, ^22^, ^23^, ^25^ ^]^

Here, we show for the first time that AS derived from the nigrostriatal system communicate via a population of vesicles enriched in sEVs, in line with the presence of exosomes and small microvesicles. Moreover, we elucidated specific EV properties according to the brain region of origin, both in terms of secretion rate and functions. Interestingly, the EV secretion rate was specific for each brain area, with VMB‐AS releasing a higher number of vesicles compared to STR‐ and ΔVS‐AS (i.e., AS from brains depleted of VMB and STR), which showed a similar rate of secretion. Also, we found that CCL3 played a critical role in the regulation of EV production, with only VMB‐AS secreting more vesicles in response to the chemokine. This AS activation strategy stems from a recent discovery made by our research team, identifying a 6‐fold upregulation of CCL3 in the VMB of PD mice, in vivo, during nigrostriatal degeneration and self‐recovery, whereby reactive AS were defined as the key components of DAergic neurorescue pathways against MPTP/MPP^+^ injury.^[^ ^94^ ^]^ In contrast, CCL3 did not increase EV secretion from STR‐ and ΔVS‐AS. However, our expression analysis of the main CCL3 receptors (i.e., Ccr1 and Ccr5), suggests that the nigrostriatal system (both VMB‐ and STR‐AS) had a specific potential to respond to the chemokine, while the expression of Ccr1 and Ccr5 in ΔVS‐AS was ≈100 times lower. Indeed, STR‐AS also responded to CCL3, but by extruding more membrane protrusions rather than increasing EV secretion. Therefore, in both nigrostriatal regions, CCL3 was able to influence the dynamics of cell membranes, in line with the membrane remodeling abilities of this chemokine.^[^ ^104^, ^105^ ^]^

Our data showing differential intrinsic and extrinsic responses of VMB‐ and STR‐AS fit with the reported high level of AS heterogeneity in the CNS, whose regulatory mechanisms (e.g., transcriptional versus epigenetic programs) remain as outstanding open questions for the field.^[^ ^77^, ^129^, ^133^, ^134^ ^]^ Indeed, the molecular machinery which orchestrates the distinct EV secretion rates—according to the brain region and the specific exposure to inflammatory triggers—needs to be further elucidated. Also, these findings call for a deeper understanding of the functional implications of such a specific response to microenvironmental cues between VMB (where the DAergic neurons reside) versus STR (where they project) for the pathogenesis of PD and, eventually, for the development of new therapeutic avenues. In fact, while a plethora of studies has identified AS harmful factors, little is known about both the mechanisms driving the induction of pro‐reparative states and their cellular/molecular effectors.^[^ ^135^, ^136^ ^]^

Initially identified as possible neurodegeneration's Trojan horse, AS‐EVs recently emerged as important mediators of “beneficial messages” to target neurons.^[^ ^137^ ^]^ However, regional differences were not taken into account, since AS‐EV preparations were mostly derived from the cerebral cortex, whole brain, or immortalized glioma cells. Also, several protocols for target neuronal cultures and/or neuron differentiation/enrichment have been reported, which make it difficult to draw firm conclusions.^[^ ^138^, ^139^, ^140^, ^141^ ^]^

Here, we used nigrostriatal AS‐EVs to investigate their specific functional roles when transferred to RA‐differentiated versus undifferentiated SH‐SY5Y cells. We showed that the EV uptake for both target cell types was similar, regardless of the regional identity/treatment of donor astrocytes. In spite of this homogeneity, we uncovered relevant functional differences in terms of their neuroprotective potential. First, differentiated SH‐SY5Y cells were exposed to two distinct sources of toxicity—H_2_O_2_ and MPP^+^—mimicking oxidative stress and mitochondrial dysfunction found in PD. Intriguingly, we found that depending on the challenge used, AS‐EVs differentially mediated neuroprotection on these target cells. In particular, both VMB‐ and STR‐AS secreted EVs able per se to counteract the cell death induced by H_2_O_2_. However, EVs derived from CCL3‐treated astrocytes showed a higher efficacy in preventing the activation of caspase‐3 in differentiated SH‐SY5Y cells, by mechanisms that warrant further investigation. Importantly, the treatment with CCL3 directly on target neurons was not able to recapitulate the neuroprotective effects of AS‐EVs. This novel finding sheds light on the mechanism of chemokine‐mediated neuroprotection previously documented for nigrostriatal AS,^[^ ^94^ ^]^ and further implicates a relevant role played by the inflammatory microenvironment in amplifying the “beneficial” AS‐mediated neuroprotection. It is important to note in this respect, that, depending on the context, chemokines can trigger either harmful or protective effects.^[^ ^91^, ^92^, ^94^ ^]^ In fact, our previous in vitro studies showed that AS exposure to CCL3 (but not to TNF‐ *α* or IL‐1*β*): i) reverted aging‐induced loss of AS neuroprotective properties against MPP^+^ cytotoxicity;^[^ ^43^ ^]^ ii) promoted neuroprotection and DAergic neurogenesis from adult midbrain NSCs;^[^ ^94^ ^]^ and iii) reverted the aged‐AS to a pro‐regenerative state.^[^ ^96^ ^]^ On the contrary, the harmful microglial environment inhibited subventricular zone NSCs, further emphasizing the capacity of chemokine‐activated AS in promoting DAergic neuron plasticity.^[^ ^44^, ^45^, ^94^, ^95^ ^]^

Considering AS protective response to basal ganglia injury,^[^ ^41^, ^42^, ^43^, ^45^, ^50^, ^94^, ^142^ ^]^ we also used MPP^+^—a well‐recognized neurotoxin recapitulating Parkinsonian symptoms^[^ ^117^, ^143^, ^144^, ^145^ ^]^—to investigate the ability of AS‐EVs to target mitochondrial function. MPP^+^ is sequestered in mitochondria where it selectively inhibits CI. Indeed, the well described deficiency of mitochondrial CI activity in the SNpc of patients with sporadic PD accounts for the majority of neuronal loss.^[^ ^68^ ^]^ We found that a preventive treatment with AS‐EVs from both VMB and STR efficiently restored CI activity in neuronal cells, severely affected by the toxin treatment. On the other hand, only VMB‐AS‐EVs fully preserved mitochondrial functionality, as demonstrated by the analysis of O_2_ flows devoted to ATP synthesis. In fact, MPP^+^ compromised the net respirations via a general reduction of the inner mitochondrial membrane (IMM) integrity,^[^ ^106^ ^]^ a critical feature for the maintenance of the proton gradient and therefore essential for the ADP phosphorylation process. In MPP^+^‐injured neurons, a part of the gradient was dissipated, by‐passing ATP synthase, and the positive effect exerted on CI was either nullified or not culminated in ATP production, as in STR‐AS‐EVs treated cells. Different intriguing factors may contribute to this novel distinct effect of VMB‐ versus STR‐AS‐EVs. Again, this specificity may depend on the particular brain area facing region‐specific neuronal vulnerabilities and/or specific tasks. For example, in the VMB, SNpc neurons are selectively vulnerable to mitochondrial CI inhibitors, versus the exquisite and “mysterious” sensitivity of STR neurons to succinate dehydrogenase (SDH, mitochondrial complex‐II) inhibitors, such as the plant‐derived mitochondrial toxin, 3‐nitropropionic acid, causing striatal damage reminiscent of Huntington's disease.^[^ ^146^ ^]^ Also, besides neuroinflammation, a peculiar striatal AS vulnerability to metabolic impairments, protein aggregation, and mitochondrial instability^[^ ^88^, ^146^, ^147^, ^148^, ^149^ ^]^ may at least in part explain this lack of “full beneficial response” observed with STR‐AS‐EVs. Further studies are needed to verify whether AS‐EV heterogeneity—in VMB versus STR—can be the result of intrinsic regional differences and/or it might be promoted by external factors present in the microenvironmental milieu. Indeed, a key aspect to be deeply explored will involve the nature of AS‐driven EV cargoes and their possible link with the mechanism(s) regulating the selection/trafficking of specific effectors toward EVs. A very long list of molecules (DNA, RNAs, proteins, lipids, metabolites, etc.) have been identified over the years within EVs, whose relative abundance changes based on the identity of the donor cell and in response to specific stimuli. Our data claim for a comprehensive multi‐omics profiling of AS‐EVs to clarify how: i) the regional identity of donor astrocytes may impact on the composition of vesicles; and ii) the relationship between potential EV‐shuttled candidates and key mitochondrial pathways in neuronal target cells. Even if we did not observe an enrichment of SDHA in AS‐EVs (see Figure 2D), different mitochondrial components may be transferred via astrocyte‐derived vesicles. Several recent reports indicate the presence of both mtDNA and fully active mitochondrial proteins, from all of the five respiratory complexes, associated with EVs, including ATP synthase, cytochrome c oxidase subunits and others.^[^ ^150^, ^151^ ^]^ However, the mechanisms by which these proteins may play a functional role in recipient cells is not fully understood. Other findings suggest the possibility that mitochondrial‐derived vesicles (MDVs)—involved in the mitochondrial quality control (MQC) system—may be rearranged within the multivesicular bodies and released in the microenvironment as exosomes.^[^ ^152^ ^]^ Interestingly, a lower secretion of MDV‐derived proteins was detected in sEVs from serum samples of PD patients versus controls, suggesting that the mitochondrial quality control (MQC) flux is impaired in PD.^[^ ^153^ ^]^ The presence of specific ncRNAs (e.g., miRNAs, tRNA‐derived fragments) and transcription factors (as mRNA or protein) within VMB versus STR AS‐EVs—able to differently regulate mitochondrial pathways in target cells—add further layers of complexity to the AS‐neuron crosstalk.^[^ ^154^, ^155^, ^156^ ^]^ Indeed, understanding the relative contribution of each potential candidate responsible for distinct functional effects on target cells remains a challenge for the field. Also, the way(s) used by AS‐EVs to interact with target cells need(s) to be further characterized.

Remarkably, the positive effects exerted by AS‐EVs were observed only when EVs were isolated from the ACM. ACM per se still contains EVs, although at a concentration approximately 50‐fold lower in comparison to the purified vesicles. This may explain the lack of any significant rescue of mitochondrial parameters when ACM was used to challenge MPP^+^ toxicity. Also, the fact that the SNT (ACM depleted of EVs) did not show any protective effect demonstrate that, in our hands, the ultracentrifugation process did not damage the vesicles, further excluding possible EV leakage.^[^ ^126^ ^]^ Although we cannot rule out the possibility that other molecules might be co‐purified with EVs, the lack of intracellular proteins—used to denote EV purity—in our vesicle preparations indicate that the AS‐EV functions are most likely due to EVs.

Finally, we showed that the same vesicle samples applied to undifferentiated SH‐SY5Y cells—exposed to either H_2_O_2_ or MPP^+^—did not exert any neuroprotective effects, in contrast to their differentiated counterparts. This specificity in the AS‐EV responsiveness points to a greater protective activity toward the dopamine neuronal phenotype, which merits further investigations. Retinoic acid (RA) is recognized to induce a neuronal differentiation program in SH‐SY5Y cells which allows the development of a predominantly mature DAergic‐like neurotransmitter phenotype.^[^ ^157^ ^]^ Accordingly, in our experimental conditions, we found that in comparison with undifferentiated cells, differentiated SH‐SY5Y displayed: i) neuron morphological and neuronal differentiation markers; and ii) a higher expression of TH (the rate limiting step in dopamine biosynthesis).^[^ ^106^ ^]^

Our data then support the notion that characteristics of AS dictated by their regional identity play important roles in modulating specifically DAergic neuron vulnerability. In fact, nigrostriatal AS display unique features, being sensitive to environmental stressors and PD neurotoxins, ageing and neuroinflammatory challenges.^[^ ^73^, ^88^, ^90^, ^147^ ^]^ Hence, under neurodegenerative conditions, many adaptive changes occur within the astroglial compartment, aimed to improve mitochondrial performance, to provide neurotrophic support, and/or to activate adult neurogenesis.^[^ ^44^, ^45^, ^72^, ^73^, ^89^, ^94^ ^]^

Overall, it seems tempting to suggest the involvement of EVs in the AS‐neuron crosstalk, in a region‐specific and context‐dependent way. More work is needed to clarify to what extent AS‐EVs contribute to AS neuroprotective effects highlighted in experimental PD models.^[^ ^158^ ^]^

This knowledge will be crucial to fully understand the functions of AS‐EVs, thus facilitating the diagnosis of CNS diseases and the identification of vesicle therapeutic potential.

## 4 Conclusion

This study provides, for the first time, an in‐depth phenotypic and functional characterization of AS‐EVs from the two main brain regions affected in PD, that is, the VMB and the STR. We demonstrate that AS from both areas produce a population of vesicles highly enriched in sEVs (≈70 nm). The EV secretion rate is specific for each brain region, with VMB astrocytes releasing more EVs per cell compared to the STR. Notably, only VMB‐AS responded to CCL3 chemokine by producing more EVs, while STR‐AS undergo plasma membrane modifications, in the absence of any effect due to cellular viability or proliferation.

Next, we showed the functional implications of these nigrostriatal‐specific differences in AS‐EV secretion, in the context of neurodegeneration. Indeed, only CCL3‐stimulated AS‐EVs are able to fully protect differentiated SH‐SY5Y cells from H_2_O_2_‐induced apoptosis. On the other hand, only VMB‐derived EVs ameliorated ATP production in the same cells injured with MPP^+^, further supporting the importance of the brain region for the accomplishment of specific functions within the brain. However, vesicles obtained from the same brain regions were not able to protect the undifferentiated SH‐SY5Y cells, thus adding a further layer of complexity to the neuroprotective program dictated by AS‐EVs.

In conclusion, our results highlight a novel role for AS‐EVs in the propagation of specific intercellular signaling, with region‐specific and inflammatory‐dependent functional implications in targeting neuroprotection. Our data further unveil the multiple levels of interaction that are established between different types of cells populating the brain. In the long term, patient‐tailored AS‐EV treatments aimed to prevent disease progression and to promote neurological recovery may be foreseen, with implications for both the etiopathology and the treatment of PD, and other NDs.

## 5 Experimental Section

### Primary Astrocyte Cultures and Treatments

Wild type C57BL/6 animals were purchased from Charles River (animal experiments were approved by the Italian Ministry of Health authorization number 442/2020‐PR). Primary astroglial cell cultures were prepared as described in Ref. [94]. Briefly, AS were obtained from mice at postnatal days P2‐P4 and isolated from VMB and STR brain regions, and from brains depleted of these two regions (ΔVS). AS were cultured in DMEM (1 g L^−1^ glucose, Sigma Aldrich, D6046) supplemented with 10% FBS (Biowest, S1810), 2 mm L‐glutamine (Sigma Aldrich, G7513), 2,5 µg mL^−1^ amphotericin B (Sigma Aldrich, A2942), and 1% penicillin/streptomycin (Sigma Aldrich, P0781) at 37 °C and 5% CO_2_ for 13–17 days in 10 cm dishes specific for primary cultures (Corning, 353 803). Loosely adherent microglial cells were then removed by shaking. Cells were washed with sterile PBS 1× and allowed to grow for another two days or reseeded onto glass coverslips in 24‐well plates for immunofluorescence (IF) analyses, or in 96‐well plates for viability and cytotoxicity analyses. Cells were washed and then treated or not with the CCL3 300 ng mL^−1^ (R&D, 450MA050), in DMEM medium supplemented with 10% FBS depleted of exosomes (System Biosciences, EXO‐FBS‐250A‐1). CCL3 concentration was based on previously published time‐course and dose‐response studies.^[^ ^43^, ^94^, ^96^, ^102^ ^]^ Cells were maintained in this medium for 24 h before supernatant collection for EV purification.

For IF analyses, AS were labelled with rabbit anti‐GFAP antibody (Dako, Z0334), while microglial cells were stained with goat anti‐Iba1 antibody (Novus, NB100‐1028). AS proliferation was evaluated by 5‐Bromo‐2′‐deoxyuridine (BrdU) incorporation assay. The day before fixation, BrdU 5 µm (Sigma Aldrich, 19–160) was added to cells for 24 h. Proliferative cells were stained with mouse anti‐BrdU antibody (Sigma Aldrich, B8434). Donkey Alexa fluor secondary antibodies were used, and nuclei were stained with DAPI (Sigma Aldrich, 32670–5MG‐F). IF images were acquired using a Leica microscope (DM5500) and analyzed with Fiji Image J software 1.51n.

For cytotoxicity analysis, 10 µL of AS supernatants were collected and analyzed by LDH‐Cytotoxicity Assay Kit (Fluorometric) (Abcam, ab197004), following the instruction provided by the kit. For viability analysis, CellTiter Blue reagent (Promega, G8080) (diluted 1:4 with PBS 1×) was added to each well of 96 well plates and incubated at 37 °C for 4 h. Then, for both kits, the fluorescent signal was measured by Varioskan flash plate reader (Thermo Fisher).

RNA was isolated from AS using the miRNeasy Mini Kit (Qiagen, 217 004). Total RNA quantity and purity were assessed with the NanoDrop ND‐1000 instrument (Thermo Scientific) and cDNA synthesis was performed using the High‐capacity cDNA reverse transcription kit (Applied Biosystem, 4 368 814). Gene expression was studied via qPCR with PowerUp SYBR Green Master Mix (Applied Biosystem, A25742), using the following primers:

Ccr1‐forward: 5′‐AGGTTGGGACCTTGAACCTTG‐3′,
Ccr1‐reverse: 5′‐ACAGTGAGTCTGTGTTTCCAGA; and
Ccr5‐forward: 5′‐TGAGACATCCGTTCCCCCTA ‐3′,

Ccr5‐reverse: 5′‐GCTGAGCCGCAATTTGTTTC‐3′. mRNA levels were normalized relative to Gusb: Gusb‐forward: 5′‐CCGACCTCTCGAACAACCG‐3′, Gusb‐reverse: 5′‐GCTTCCCGTTCATACCACACC‐3′. Samples were tested in triplicate on a QuantStudio 3 Real‐Time PCR System (Applied Biosystem) and expressed as ΔCt.

### Scanning Electron Microscopy (SEM) Processing

Cells were fixed in 3% Glutaraldehyde (Sigma Aldrich, G5882) for 1 h. Samples were then post‐fixed in 1% osmium tetroxide for 45 min at 4 °C. Samples were washed with deionized water and partially dehydrated in increasing concentrations of ethanol up to 100% ethanol. Subsequently, critical point drying and sputtering with gold/palladium alloy was performed at the Central Service for Experimental Research of the University of Valencia. SEM images were obtained on a Hitachi S4800 microscope.

### Histological Processing

Cells were fixed in 3% glutaraldehyde for 1 h, then they were post fixed with 2% osmium tetroxide (Electron Microscopy Sciences) for 2 h. Sections were then washed in deionized water, and partially dehydrated in 70% ethanol. Afterward, the samples were contrasted in 2% uranyl acetate (Electron Microscopy Sciences) in 70% ethanol for 2 h at 4 °C. The samples were further dehydrated and infiltrated in Durcupan ACM epoxy resin (Sigma) at room temperature overnight, and then at 60 °C for 72 h. 1.5 µm sections were obtained using an Ultracut UC7 ultramicrotome (Leica Biosystems). Sections were stained with 1% Toluidine Blue. Images were taken with an i80 Nikon Microscope.

### EVs Isolation and Characterization

AS supernatants were collected and immediately centrifuged at 1000 × *g* at 4 °C for 15 min in order to pull down residual cells/cell debris. Next, the supernatants were subjected to ultracentrifugation in a Sorvall WX100 (Thermo Scientific). The first ultracentrifugation was performed at 100 000 g at 4 °C for 75 min, in ultra‐cone polyclear centrifuge tubes, each containing the supernatant deriving from ≈15 × 10^6^ astrocytes (Seton, 7067), using the swing‐out rotor SureSpin 630 (k‐factor: 216, RPM: 23 200). Then the pellet was washed with cold PBS 1× and ultracentrifuged again at the same speed for 40 min in thick wall polycarbonate tubes (Seton, 2002), using the fixed‐angle rotor T‐8100 (k‐factor: 106, RPM: 41 000). The resulting pellets, containing AS‐EVs, were resuspended in PBS 1× (for NTA, EM and functional experiments), in RIPA buffer (for WB characterization), or in Diluent C (for PKH26 staining).

### Nanoparticle Tracking Analysis (NTA)

AS‐EVs were diluted in PBS 1× and analyzed for particle size distribution and concentration on a Nanosight NS500 (Malvern Instruments Ltd, UK) fitted with an Electron Multiplication‐Couple Device camera and a 532 nm laser. The sample concentration was adjusted to 10^8^–10^9^ particles/mL and measurements were performed in static mode (no flow) at an average temperature of 21 ± 1 °C. A total of 3 to 5 videos of 60 s were recorded for each independent replicate, loading a fresh sample for each measurement. Videos were processed on NTA software v3.2 and a detection threshold of 8 was used. The remaining settings were set to automatic. Total particle concentration for each EV sample was determined by NTA and used to calculate the number of EVs released per 10^6^ cells.

### EV Negative Staining for Transmission Electron Microscopy (TEM)

AS‐EVs were fixed with 2% paraformaldehyde (PFA) (Sigma Aldrich, P6148) in PBS 1× for 30 min. 200 mesh formvar and carbon coated nickel grids were glow‐discharged to make the surface grid hydrophilic. Fixed samples were placed on the grids for 7 min, samples were washed with ultrapure water and stained with 2% uranyl acetate for 7 min and examined at 80 kV on a FEI Tecnai G2 Spirit (FEI Company, Hillsboro, OR) transmission electron microscope equipped with a Morada CCD digital camera (Olympus, Tokyo, Japan). To obtain the number of vesicles in EM, 10 random  fields (from 60 000 × magnification) were counted, each from a different square of the 200‐mesh grid, per each condition.^[^ ^159^ ^]^ The results were normalized taking into account the following parameters: the number of starting cells, the resuspension volume after ultracentrifugation, the volume used in the microscope grid, and the area (µm^2^) of each field in the grid.

### EV Immunogold Labelling for Transmission Electron Microscopy (TEM)

To increase the hydrophobic properties of the grids 200 mesh formvar and carbon coated nickel grids were glow‐discharged. Grids were placed on a 10 µL drop of each sample for 7 min and washed with PBS 1×. Nonspecific reactions were avoided using blocking solution containing 0.3% BSA for 30 min. Then, samples were washed in 0.1% BSAc (Aurion, Wageningen, the Netherlands) in PBS 1×. The samples were incubated in 10 µL of 1:50 primary antibody (rat anti‐CD9 or rat anti‐CD63, see **Table** **1**) in 0.1% BSAc (Electron Microscopy Sciences) for 1 h. After, the samples were washed in 0.1% BSAc and incubated in 1:20 goat anti‐rat 6 nm gold particles (Abcam, ab105300) in 0.1% BSAc for 1 h in the dark. Grids were rinsed with 0.1% BSAc and fixed with 2% glutaraldehyde for 5 min and washed with ultrapure water. Finally, negative staining with 2% uranyl acetate was performed for 5 min. The samples were examined at 80 kV on a FEI Tecnai G2 Spirit (FEI Company, Hillsboro, OR) transmission electron microscope equipped with a Morada CCD digital camera (Olympus, Tokyo, Japan).

### Western Blotting

AS and EVs extracts were processed as in Ref.[97, 98]. Briefly, AS and EVs were lysed in RIPA buffer (10 mm Tris HCl pH 7.2 (Fisher Scientific, BP152); 1% sodium deoxycholate (Sigma Aldrich, 30 970); 1% Triton X‐100 (Sigma Aldrich, T8787); 0,1% (for cells) or 3% (for EVs) SDS (Sigma Aldrich, 71 736); 150 mm NaCl (Sigma Aldrich, S7653); 1 mm EDTA pH 8 (VWR chemicals, E177‐100ML); 1 mm phenylmethanesulfonyl fluoride solution (PMSF, Sigma Aldrich, 93 482); 1× Complete Protease inhibitor cocktail (Roche, 0 469 311 6001), 1× Halt Phosphatase inhibitor cocktail (Thermo Fisher Scientific, 78 420)), and protein concentration was measured with *DC* Protein Assay (Biorad, 500‐0116), using BSA (Pierce, 23 210) as standard (AS‐EV protein yield: 0.5–1.5 µg/10^6^ cells). The same amount of cell or EV lysates was then loaded into 4–12% Bis‐Tris plus gels (Invitrogen, NW04125BOX) in reducing or non‐reducing conditions. Afterward, proteins were transferred onto PVDF membrane. All primary and secondary antibodies are listed in Table 1.

**Table 1:** List of antibodies used in WB

Antibody	Dilution	Brand	Catalog number
Rat monoclonal anti‐CD63	1:5000	MBL	D263‐3
Rat monoclonal anti‐CD9	1:5000	BD Pharmigen	553758
Mouse monoclonal anti‐Pdcd6ip	1:500	BD transduction lab	611620
Mouse monoclonal anti‐SDHA	1:1000	Abcam	ab14715
Rabbit polyclonal anti‐Canx	1:10 000	Abcam	ab22595
Mouse monoclonal anti‐GM130	1:1000	BD transduction lab	610823
Mouse monoclonal anti‐*β*‐actin	1:10 000	Sigma Aldrich	A1978
HRP‐conjugated anti‐mouse secondary antibody	1:10 000	Dako	P0447
HRP‐conjugated anti‐rabbit secondary antibody	1:10 000	Invitrogen	31460
HRP‐conjugated anti‐rat secondary antibody	1:10 000	Invitrogen	31470

### SH‐SY5Y Culture, Differentiation, and Treatments

SH‐SY5Y cells were purchased from ICLC (Interlab Cell Line Collection, accession number ICLC HTL95013; obtained from depositor European Collection of Authenticated Cell Cultures [ECACC]) and cultured and differentiated as described in Ref. [106]. Briefly, cells were maintained in MEM/F12 medium (Biochrom GmbH, F0325 and Sigma Aldrich, N4888). For cell differentiation, MEM/F12 was replaced with DMEM/F12 and 10 µm retinoic acid (Sigma Aldrich, R2625), and cultivated for 8 days with gradual serum deprivation until 0.5% FBS. At the end of differentiation, cells were detached and seeded at the density of 3 × 10^5^ cells/cm^2^ in 12‐well (for IFC analysis, see Section 5.10 for EV labeling), 96‐well (for dose response curve), 24‐well (for c‐Casp‐3 IF staining), or 6‐well (for HRR analysis, see Section 5.11) plates. For all the experiments where EVs were applied on target cells, the authors used the ratio 5:1 (i.e., EVs derived from five AS used to treat one SH‐SY5Y cell).

For IFC analysis, labelled AS‐EVs were applied on differentiated and undifferentiated SH‐SY5Y cells (see below) seeded on 12 well plates. Internalization was evaluated at different time points (i.e., 2, 6, and 24 h) at 20× magnification by using the Amnis FlowSight Imaging Flow Cytometer (Luminex). At the end of each time point, cells were trypsinized and collected in 1 mm EDTA + 1% BSA. For all passages cells were kept on ice. Fluorescence intensity of PKH26 was measured by using 488 nm laser. Flow cytometric gating was used to select focused single cells and the mean fluorescence intensity of treated cells was compared with that of untreated cells. For normalization, the authors analyzed the first 1000 single cells, in order of acquisition, with an optimal focus, using IDEAS software version 6.2 183.0 (Amnis, part of Luminex).

Two dose‐response curves, one for H_2_O_2_ (Sigma Aldrich, H1009) and one for MPP^+^ (Sigma Aldrich, D048), were performed at 24 h, using CellTiter Blue (Promega, G8080), as described in the Primary Astrocyte Cultures and Treatments section.

For IF, cells were seeded on poly‐L‐lysine coated glass coverslips. After two days, EVs were applied on target cells. As a control, the vesicles eventually present as contaminants in the medium used to culture AS (cont‐EVs) were also tested following the same experimental steps used for AS‐EVs. Following ultracentrifugation, cont‐EVs were resuspended in PBS 1× and used to treat SH‐SY5Y maintaining the same ratio with the starting volume of medium, as for the purification of AS‐EVs. 6 h later, cells were treated with 35 µm H_2_O_2_ for a further 24 h. Coverslips were fixed with 4% PFA and stained with rabbit polyclonal anti‐c‐Caspase‐3 (Cell Signaling, 9664) primary antibody and with mouse monoclonal anti‐map2 primary antibody (Merck Millipore, MAB3418). The secondary antibodies used were the anti‐Rabbit Alexa Fluor 546 (Thermo Fisher Scientific, A10040), and the anti‐Mouse Alexa fluor 488 secondary antibodies (Thermo Fisher Scientific, R37114). Nuclei were counterstained with DAPI. IF images were acquired using a Leica microscope (DM5500) and analyzed with Fiji Image J software. The intensity of the c‐Casp‐3 signal was measured by using the following steps in ImageJ software: i) analyze; ii) measure; and iii) integrated density, as in Ref. [160]. Integrated density was normalized for the number of DAPI^+^ nuclei.

As a further control, the chemokine CCL3 (at 30 and 300 ng mL^−1^) was added directly to SH‐SY5Y cell cultures on 96‐well plate 6 h before H_2_O_2_ exposure. Cell viability/death was evaluated 24 h after the H_2_O_2_ treatment with CellTiter Blue and Caspase‐Glo 3/7 Assay (Promega, G8091).

Undifferentiated SH‐SY5Y cells were seeded at a density of 1 × 10^5^ cells/cm^2^ in 96, 12 and 6‐well plates. For apoptosis analysis, cells were seeded in 96‐well plates. Two days after, cells were treated with AS‐EVs, then after 6 h with 35 µm H_2_O_2_ and finally analyzed with the Caspase‐Glo 3/7 Assay after a further 24 h. For IFC and HRR analysis (see below), cells were seeded in 12 and 6‐well plates, respectively, and processed like differentiated SH‐SY5Y cells.

### EV Labelling

EV internalization was analyzed with two different approaches of labelling. First, AS were treated with the lipophilic dye PKH26 (Sigma Aldrich, MINI26‐1KT), following the protocol suggested by the manufacturer. After 3 days cells were washed, and medium changed with DMEM supplemented with 10% FBS depleted of exosomes. EVs were isolated from AS supernatants after 24 h by ultracentrifugation. The resulting EVs were applied on differentiated SH‐SY5Y cells seeded onto poly‐L‐lysine (Sigma Aldrich, P9155) coated glass coverslips in 24 well plates. Target cells were stained with *α*‐TH primary antibody (Millipore, AB152) as in Ref. [106]. Imaging was performed using the confocal laser scanning microscope Leica TCS SP8. Image acquisitions were performed through LAS X software (Leica Microsystems). Image analyses were done using the open‐source Java image processing program Fiji is Just ImageJ (Fiji). 3D reconstruction was done with the Fiji 3D Viewer dedicated plugin.

For the second approach, EVs were directly labelled with the same lipophilic dye, following the protocol suggested by the manufacturer, with some modification. Briefly, EVs derived from 90 mL of AS supernatant were ultracentrifuged, and the resulting pellets were resuspended in 0.3 mL of Diluent C plus 4 µL of dye, and incubated at room temperature for 5 min, mixing every 30 s. The labeling was quenched by adding 1% BSA in PBS 1× and again ultracentrifuged. The resulting pellet, containing the labelled EVs, were resuspended in 100 µL PBS 1×. Residual PKH26 was eliminated into the Exosome Spin Column (Thermo Fisher Scientific, 4 484 449) according to the manufacturer's recommendations. Again, eluted EVs were applied on differentiated SH‐SY5Y cells seeded onto glass coverslips in 24 well plates. At the end of the treatment cells were fixed with 4% PFA. As a control, PBS 1× with the same concentration of PKH26 dye was centrifuged under the same conditions and added to target cells. IF images were acquired using a Leica microscope (DM5500) and analyzed with Fiji Image J software 1.51n. For IFC analysis see the SH‐SY5Y Culture, Differentiation, and Treatments section.

### High‐Resolution Respirometry (HRR)

The capacity of different respiratory states in differentiated or undifferentiated SH‐SY5Y cells was assayed by High‐Resolution Respirometry (HRR) using the O2k‐FluoRespirometer (Oroboros Instruments). Cells were seeded in 6‐well plates and, after two days, AS‐EVs were applied on the top of SH‐SY5Y cells, as before. As control, 30% of ACM or supernatant (ACM after ultracentrifugation, SNT) were applied on target cells. 6 h later, cells were treated with MPP^+^ 1 mm and analyzed after further 24 h. All the experiments were performed in mitochondrial respiration buffer Mir05 (Oroboros Instrument, 60101‐01) at 37 °C under constant stirring (750 RPM). A specific Substrate‐Uncoupler‐Inhibitor Titration (SUIT) protocol was used for the determination of the O_2_ consumption in each specific respiratory state, as detailed in Ref. [106]. Briefly, respiration in the presence of endogenous substrates or ROUTINE was measured in intact cells. The mild‐detergent digitonin (Sigma Aldrich, D5628) was added at the final concentration of 4 µm in order to obtain the permeabilization of plasma membrane without compromising the mitochondrial membranes’ integrity. The O_2_ consumption after permeabilization or LEAK was determined in the presence of 5 mm pyruvate (Sigma Aldrich, P2256) and 2 mm malate (Sigma Aldrich, M1000), but not adenylates. The contribution of complex I to the OXPHOS respiration was achieved by the addition of 10 mm glutamate (Sigma Aldrich, G1626) in the presence of a saturating concentration of ADP (2.5 mm, Sigma Aldrich, 117 105). The OXPHOS respiration was then stimulated with the addition of 10 mm succinate (Sigma Aldrich, S2378). The uncoupled maximal capacity of the electron transport system (ETS) was obtained after titration with 0.5 µm of uncoupler carbonyl cyanide 3‐chlorophenylhydrazone (CCCP, Sigma Aldrich, C2759) up to the complete dissipation of the proton gradient. Finally, the residual O_2_ consumption or ROX was obtained upon addition of 2 µm rotenone (Sigma Aldrich, R8875) and 2.5 µm antimycin A (Sigma Aldrich, A8674). The O_2_ consumption in ROUTINE, LEAK, OXPHOS, and ETS capacity was corrected for the ROX. Values were then expressed as Flux Control Ratio (FCR) of the maximal respiration, using ETS capacity as a reference state.^[^ ^161^ ^]^ The O_2_ flux related to ATP synthesis was determined by correcting ROUTINE and OXPHOS for the LEAK respiration. Coupling efficiencies were calculated by correcting each state for LEAK respiration and expressing it as a percentage of the capacity in that specific state.^[^ ^161^ ^]^ Instrumental and chemical background fluxes were calibrated as a function of the O_2_ concentration using DatLab software (version 7.4.0.1, Oroboros Instruments).

### Statistical Analysis

Pre‐processing of data are described in each figure legend. The statistical analyses were performed with GraphPad Prism software (version 9.2.0). For all the analyses, differences among groups were analyzed by one‐way ANOVA followed by a Tukey's multiple comparisons test. The values are expressed as mean (± SD) and a *p* < 0.05 was accepted as significant. For IF on AS, data were obtained from *n* = 4 (for VMB‐ and STR‐AS) or *n* = 3 (for ΔVS‐AS) independent biological replicates (from 4 to 10 images for each replicate). For cell viability/cytotoxicity on AS, data were obtained from *n* = 3 independent replicates. For NTA, data were obtained from *n* = 3 independent biological replicates (a total of 3 to 5 videos of 60 s recorded for each biological replicate). For EM, data were obtained from *n* = 5 (for VMB‐ and STR‐AS) or *n* = 3 (for ΔVS‐AS) independent biological replicates (10 fields for each replicate). For qPCR, data were obtained from *n* = 3 independent biological replicates. For IFC analysis on SH‐SY5Y cells data were obtained from *n* = 3 independent biological replicates. For the dose‐response curve of H_2_O_2_ and MPP^+^, data were obtained from *n* = 3 independent biological replicates were analyzed by nonlinear regression, dose‐response‐inhibition ([Inhibitor] versus response—variable slope [four parameters]). For IF on SH‐SY5Y, data were obtained from *n* = 3 independent biological replicates (from 4 to 8 images for each biological replicate). For cell viability and apoptosis on differentiated and undifferentiated SH‐SY5Y cells, data were obtained from at least *n* = 2 independent biological replicates. For HRR measurement on differentiated SH‐SY5Y treated with AS‐EVs, the following independent biological replicates have been performed: *n* = 4 for CTRL and MPP^+^, *n* = 3 for +/− VMB‐AS‐EVs, *n* = 2 for +/− STR‐AS‐EVs. For HRR measurement on differentiated SH‐SY5Y treated with ACM/SNT, the following independent biological replicates have been performed: *n* = 3 for CTRL and *n* = 2 for MPP^+^, VMB‐ACM/SNT, STR‐ACM/SNT. For HRR measurement on undifferentiated SH‐SY5Y treated with AS‐EVs, the following independent biological replicates have been performed: *n* = 3 for CTRL and *n* = 2 for MPP^+^, VMB‐AS‐EVs, and STR‐AS‐EVs.

All relevant data of the experiments are available at the EV‐TRACK knowledgebase (EV‐TRACK ID: EV220106).^[^ ^162^ ^]^

## Conflict of Interest

A.S. is an employee of Luminex B.V., which is a subsidiary of Luminex Corporation a DiaSorin Company. Luminex Corporation is the manufacturer of the Amnis FlowSight Imaging Flow Cytometer.

## Authors Contribution

L.L., F.L.E., A.Ma., N.F., L.P.‐J., S.P., J.M.G.‐V, A.Me., B.M., and N.I. conceived and designed the experiments; L.L., F.L.E., G.P., N.T., F.P., and N.I. performed primary astrocyte cultures; L.L., F.L.E., G.P., S.V., F.P., and N.I. performed SH‐SY5Y cultures and treatments; L.L., G.P., S.V., F.P., L.P.‐J., S.P., and N.I. carried out EV isolation and labeling; L.L., F.L.E., G.P., S.V., C.T., B.M., and N.I. carried out immunofluorescence and confocal microscopy; L.L., G.P., S.V., A.S., and N.I. performed IFC analyses; L.L., G.P., S.C., and N.I. performed WB analysis; L.L., C.A.P.B., J.J.P, N.F., and N.I. performed nanoparticle tracking analysis; L.L., M.J.U.‐N., J.M.G.‐V., and N.I. performed electron microscopy analysis; L.L., A.Ma., G.P., P.R., A.Me., and N.I. performed high‐resolution respirometry analysis; L.L., F.L.E., A.Ma., M.J.U.‐N., G.P., S.V., C.A.P.B., and N.I. performed statistical analyses; L.L., A.Ma., B.M., and N.I. wrote the original draft; L.L., A.Ma., S.V., L.P.‐J., S.P., J.M.G.‐V., A.Me., B.M., and N.I. performed review and revision of the paper. All authors contributed to the preparation of the figures and to the final version of the manuscript. All authors read and approved the final manuscript.

## Supporting information

Supporting Information

Supplemental Movie 1: video showing the 3D reconstruction from all Z‐stacks for SH‐SY5Y treated with VMB‐AS‐EVs (see Figure 3A, upper panel).

Supplemental Movie 2: video showing the 3D reconstruction from all Z‐stacks for SH‐SY5Y treated with STR‐AS‐EVs (see Figure 3A, lower panel).

## Data Availability

The data that support the findings of this study are available from the corresponding author upon reasonable request.
