# Supplementary material for: Small Extracellular Vesicles Secreted by Nigrostriatal Astrocytes Rescue Cell Death and Preserve Mitochondrial Function in Parkinson's Disease
Source: Adv Healthc Mater. 2022 Aug 15;11(20):2201203. doi: 10.1002/adhm.202201203 (PMC11468249; doi:10.1002/adhm.202201203)
Supplement: Supplementary file 1 — Supporting Information [file ADHM-11-2201203-s003.pdf]

ADVANCED HEALTHCARE MATERIALS

Supporting Information

for *Adv. Healthcare Mater.*, DOI 10.1002/adhm.202201203

Small Extracellular Vesicles Secreted by Nigrostriatal Astrocytes Rescue Cell Death and Preserve Mitochondrial Function in Parkinson's Disease

Loredana Leggio, Francesca L'Episcopo, Andrea Magrì, María José Ulloa-Navas, Greta Paternò, Silvia Vivarelli, Carlos A. P. Bastos, Cataldo Tirolo, Nunzio Testa, Salvatore Caniglia, Pierpaolo Risiglione, Fabrizio Pappalardo, Alessandro Serra, Patricia García-Tárraga, Nuno Faria, Jonathan J. Powell, Luca Peruzzotti-Jametti, Stefano Pluchino, José Manuel García-Verdugo, Angela Messina, Bianca Marchetti and Nunzio Iraci**

Supporting Information

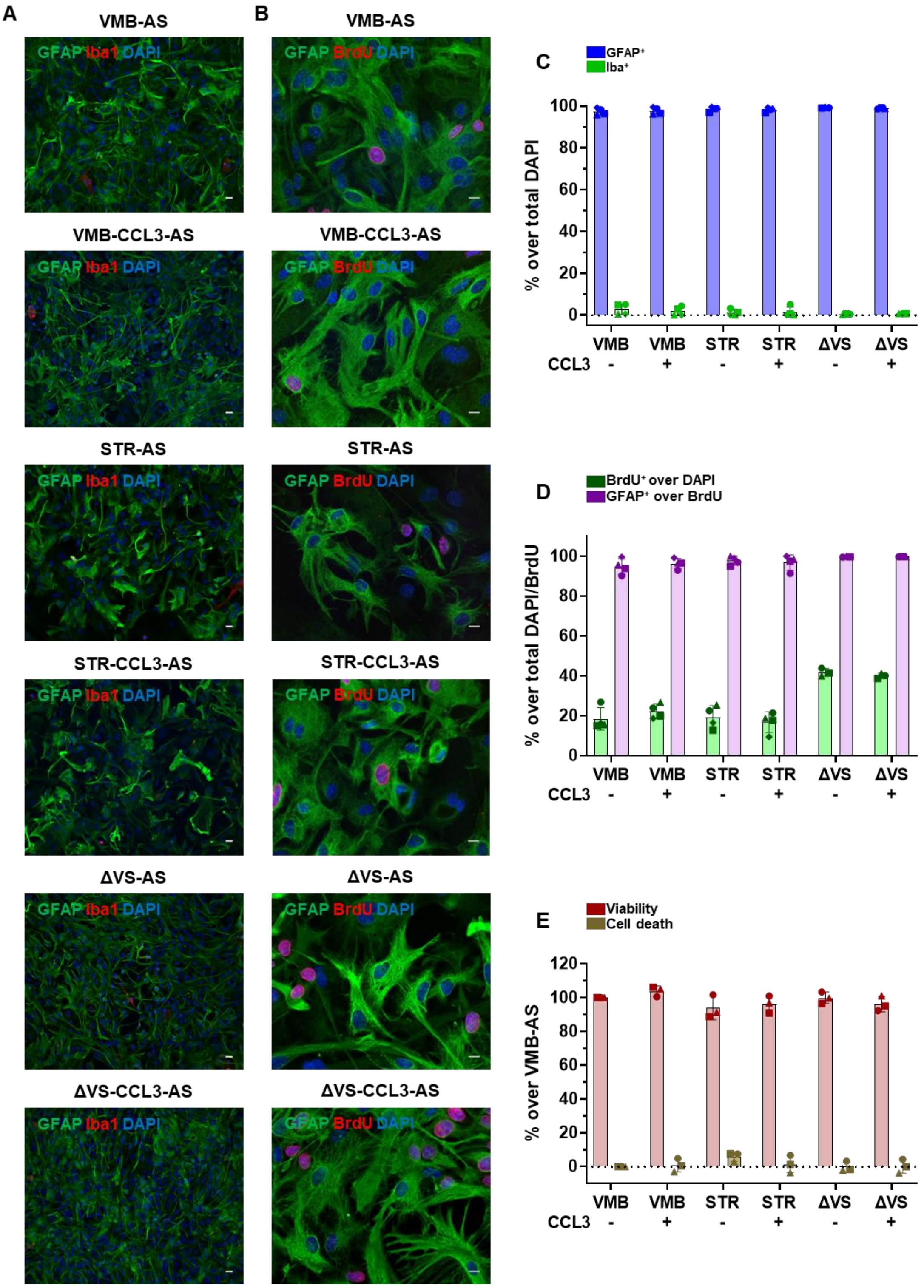

Figure S1. Characterization of AS primary cultures from VMB, STR and from VMB- and STR-depleted brain regions (ΔVS-AS), under basal and CCL3-treated conditions. A) Immunofluorescence (IF) images show the presence of AS ($GFAP^+$ cells, in green) and microglial cells ($Iba1^+$ cells, in red), with $DAPI^+$ nuclei (in blue). Scale bars: 20 µm. B) IF images show the presence of proliferative AS ($GFAP^+/BrdU^+$ cells, in green and red respectively), with $DAPI^+$ nuclei (in blue). Scale bars: 10 µm. C) Quantification of the staining in A: the number of $GFAP^+$ and $Iba1^+$ cells are normalized over total $DAPI^+$ nuclei. Data are expressed as mean ± SD from n=4

(for VMB- and STR-AS) and n=3 (for ΔVS-AS) independent experiments, indicated with different symbols. D) Quantification of the staining in B: the number of $BrdU^+$ cells are normalized over total $DAPI^+$ nuclei, while $GFAP^+$ cells are normalized over $BrdU^+$ cells. Data are expressed as mean ± SD from n=4 (for VMB- and STR-AS) and n=3 (for ΔVS-AS) independent experiments, indicated with different symbols.
E) Analysis of cell viability and death. Data are expressed as mean over VMB-AS ± SD from n=3 independent experiments, indicated with different symbols.

Table S1. Diameter values of AS-EV samples.

Diameter (nm)	VMB-AS-EVs	VMB-CCL3-AS-EVs	STR-AS-EVs	STR-CCL3-AS-EVs	ΔVS-AS-EVs	ΔVS-CCL3-AS-EVs
Minimum	28,28	22,12	24,19	24,25	18,5	23,7
Maximum	290,8	440,1	280,3	340,9	142,5	192,7
Median	63,5	59,5	64,9	59,6	53,3	54,0
Mean	72,5	68,5	75	63,5	58,7	64
Std. Deviation	14,5	14,7	7	17,8	6,8	8,5

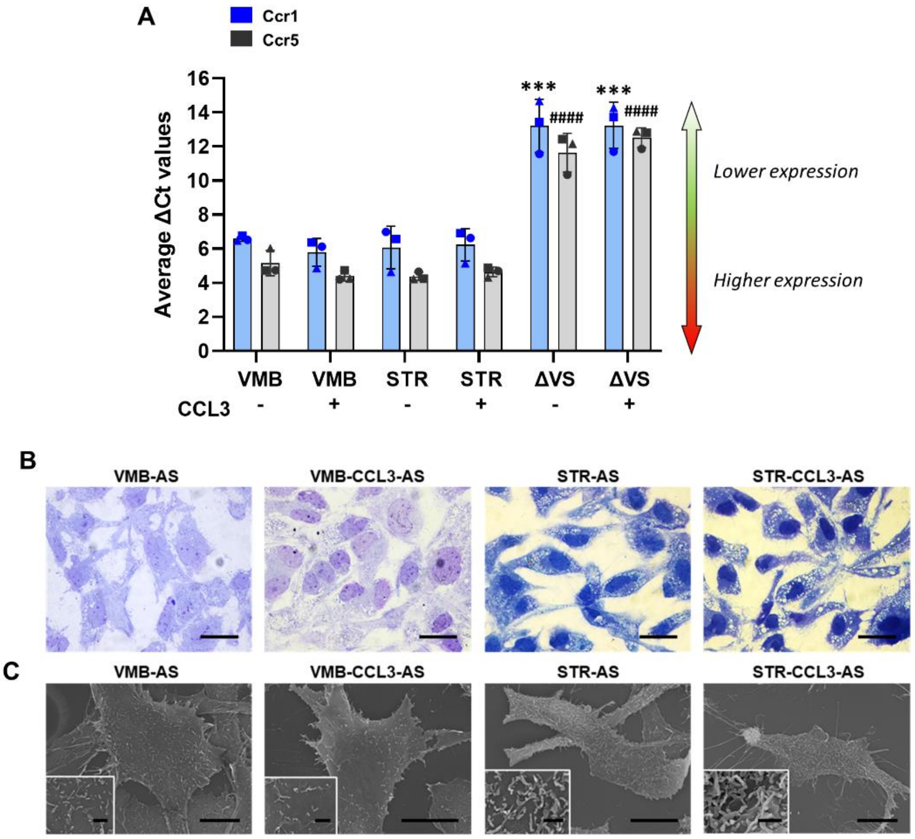

Figure S2. A) qPCR analyses of CCL3 receptors in AS, showing average ΔCt values for Ccr1 and Ccr5. Gusb was used as housekeeping gene. Data are presented as mean ± SD from n=3 independent replicates, indicated with different symbols. One-way ANOVA with Tukey's multiple comparison test shows that ΔCt values for Ccr1 in ΔVS-AS are significantly higher compared to all the other groups (∗∗∗p < 0.001); ΔCt values for Ccr5 in ΔVS-AS are significantly higher compared to all the other groups (####p < 0.0001). B) Semithin sections stained with toluidine blue show differences in the membranes of STR-AS (CCL3 vs. basal) but not in VMB. C) SEM analysis shows that STR-AS bear more irregular membrane protrusions after CCL3 supplementation. Scale bars B: 50 µm, C: 10 µm, inserts: 1 µm.

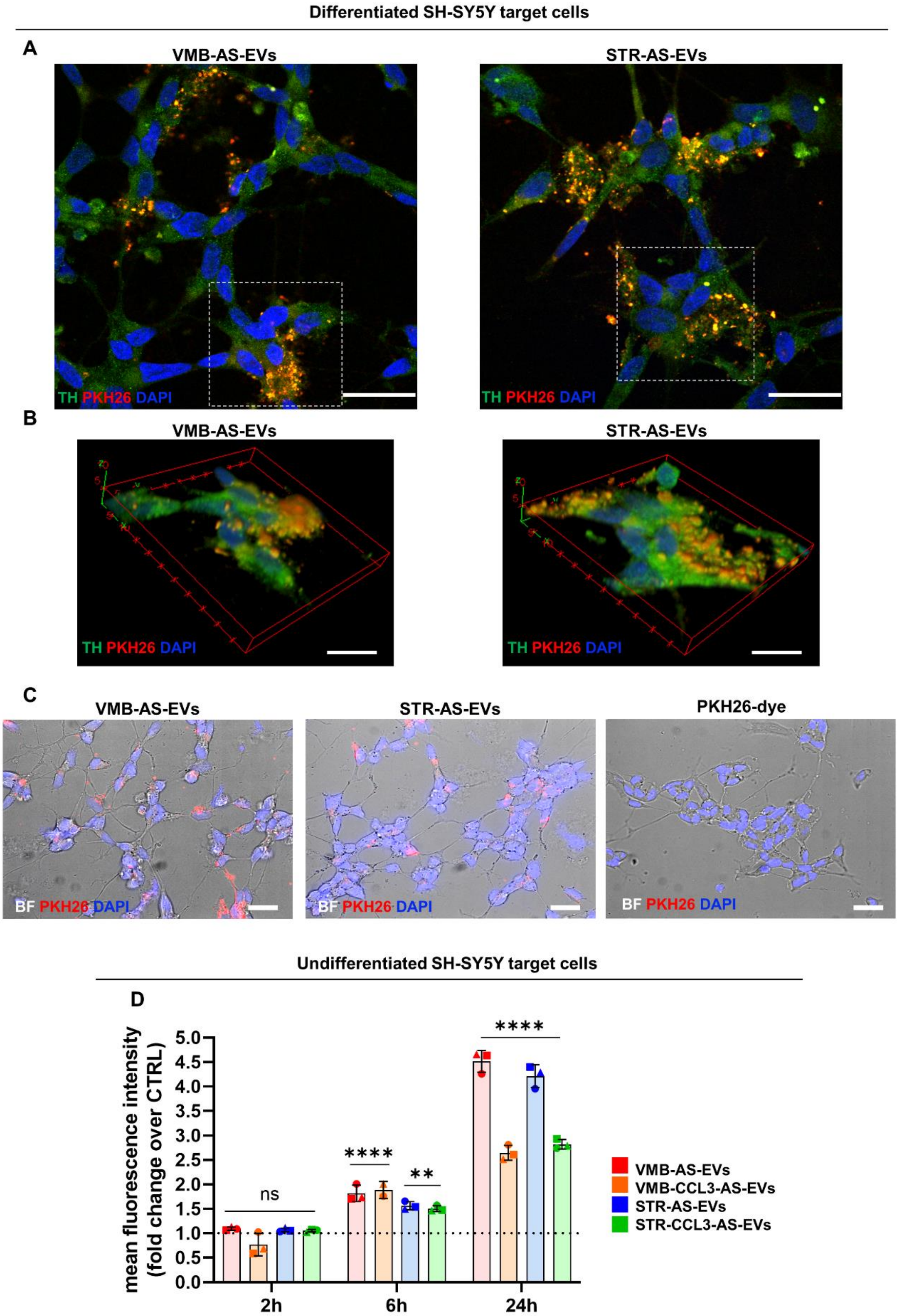

Figure S3. PKH26-labelled AS-EVs internalization by differentiated and undifferentiated SH-SY5Y cells. A) Single plan confocal images show the uptake of both VMB-AS- and STR-AS-PKH26-labelled EVs by differentiated SH-SY5Y (white dotted squares were shown as max projections in Figure 3A). Scale bar 20 µm. B) 3D reconstruction from all z stacks (see Figure 3A). Scale bars 10 µm. C) IF (in red PKH26 labelled AS-EVs and in blue DAPI counterstained nuclei) and bright field (whole cells) images of differentiated SH-SY5Y upon treatment with PKH26-labeled EVs. EVs are distributed in cell bodies and also in neurites. On the right, PKH26

dye-only were administered to target cells. Scale bars: 20 µM. D) IFC analysis of undifferentiated SH-SY5Y cells treated with PKH26-AS-EVs at different time points. Data are expressed as fold change of the mean fluorescence intensity ± SD over CTRL (dashed line at y axis =1) from n=3 independent experiment, indicated with different symbols. One-way ANOVA with Tukey's multiple comparison vs. CTRL. $**p < 0.01$, $****p < 0.0001$, ns: not significant.

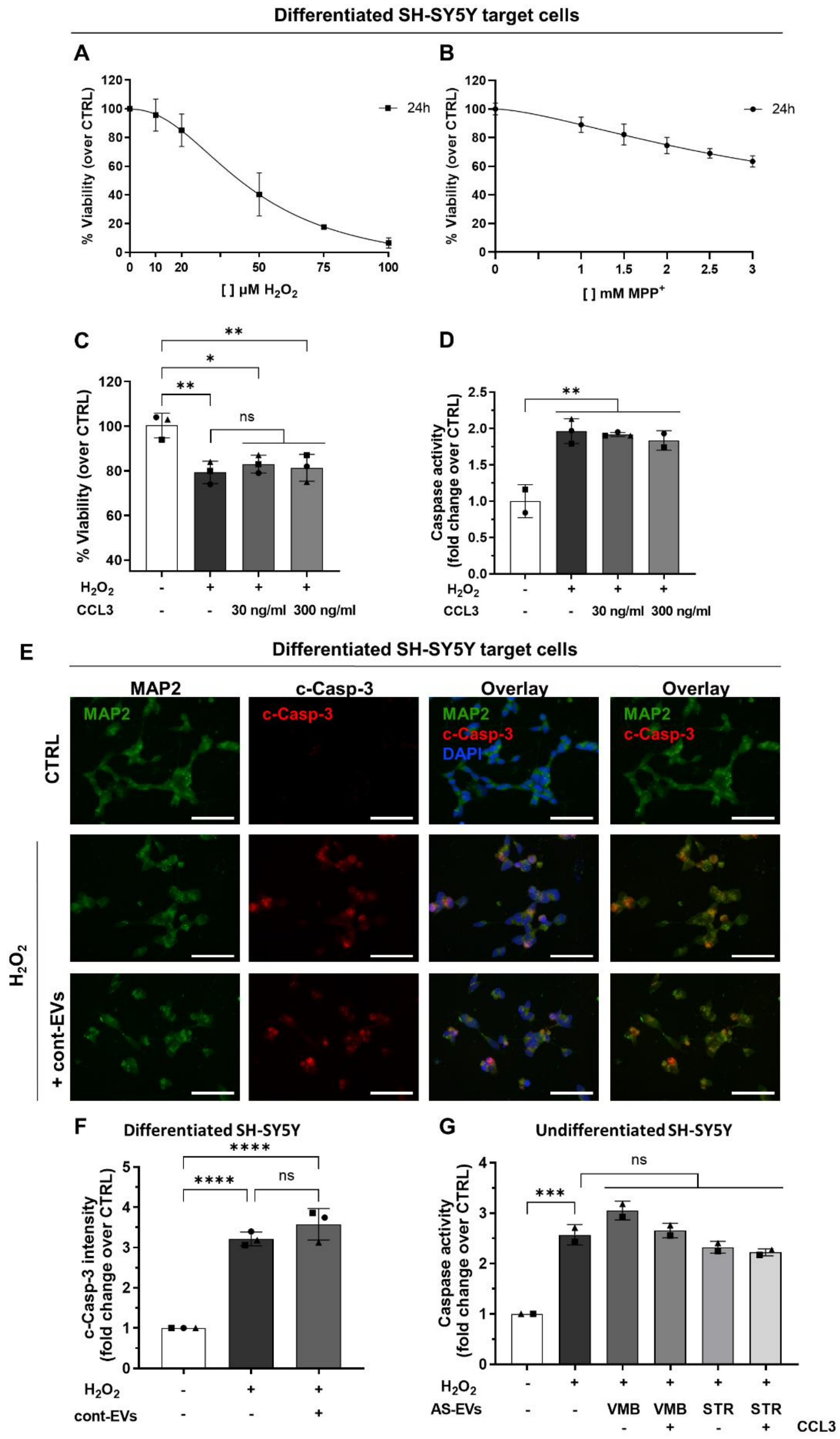

Figure S4. A) Dose response curve of H_2O_2 on differentiated SH-SY5Y cells at 24 h. B) Dose response curve of MPP^+ on differentiated SH-SY5Y cells at 24 h. C-D) Analysis of cell viability (C) and cell death (D) of differentiated SH-SY5Y neurons treated with CCL3 and challenged with H_2O_2, expressed as percentage (in C) or fold change (in D) over CTRL. Data are expressed as mean ± SD from n=3 independent replicates, indicated with different symbols. One-way ANOVA with Tukey's multiple comparison $*p < 0.05$, $**p < 0.01$ vs. CTRL,

ns: not significant. E) IF staining for MAP2 (in green), c-Casp-3 (in red) and DAPI (in blue), on differentiated SH-SY5Y exposed to cont-EVs and treated with 35 µM H_2O_2. Scale bars: 50 µm. F) Quantification of the c-Casp-3 staining in E. The fluorescent intensity values were normalized over total $DAPI^+$ nuclei. Data are expressed as mean ± SD over CTRL, set to 1 for comparison. G) Caspase 3/7 activities in undifferentiated SH-SY5Y exposed to AS-EVs (ratio 5:1) for 6 h and then treated with 35 µM H_2O_2 for 24 h. Data are expressed as mean ± SD over CTRL, set to 1 for comparison. One-way ANOVA with Tukey's multiple comparison. In (F) ∗∗∗∗$p < 0.0001$ (CTRL vs. H_2O_2 and vs. H_2O_2 + cont-EVs), ns: not significant. In (G) ∗∗∗$p < 0.001$ (CTRL vs. H_2O_2), ns: not significant.

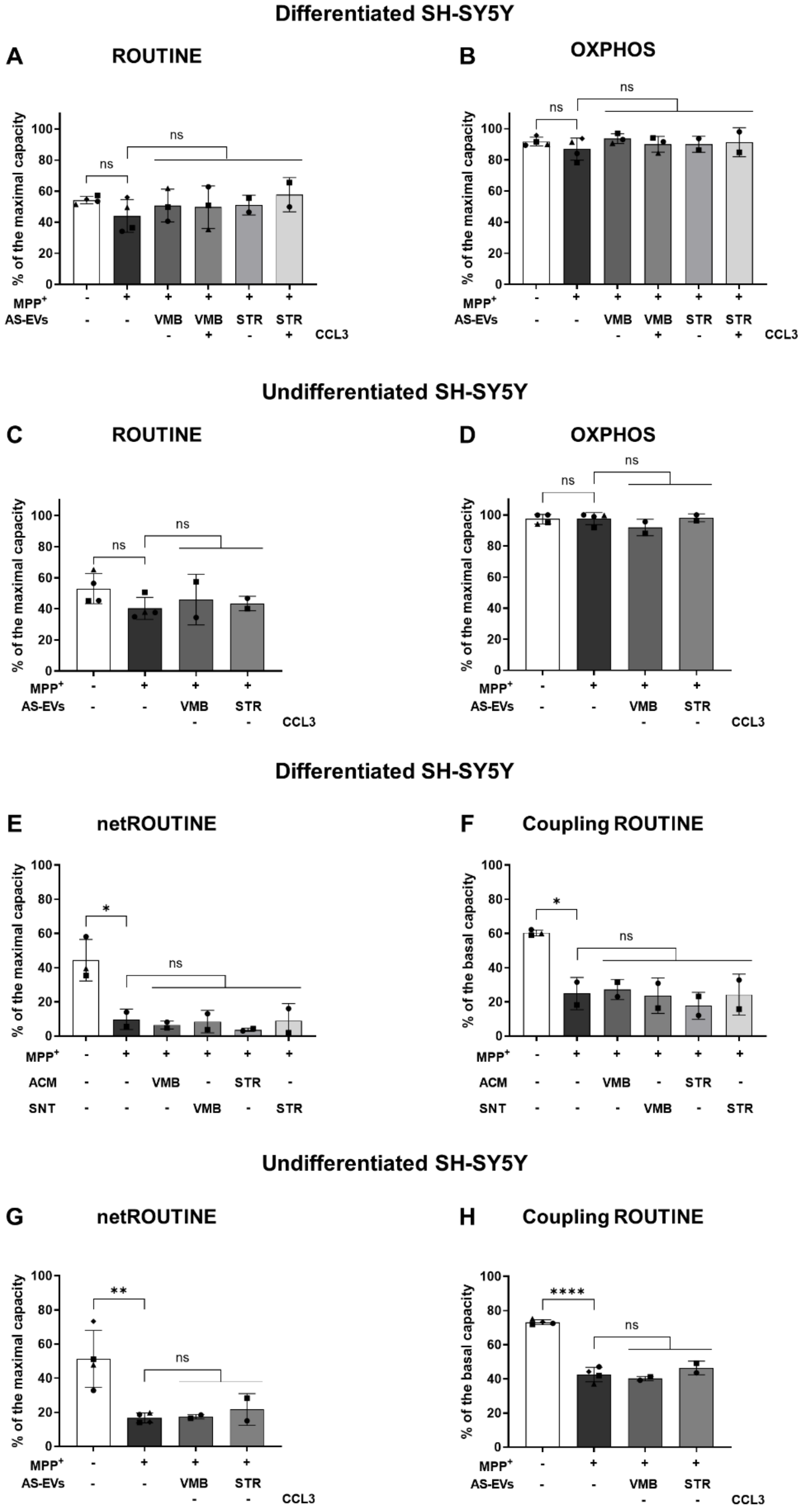

Figure S5 A-D) Analysis of O_2 flows correspondent to the main respiratory states ROUTINE and OXPHOS achieved upon different experimental conditions and/or EV treatment in differentiated (A-B) or undifferentiated (C-D) SH-SY5Y cells. MPP^+ did not affected respiration in any condition tested. All data are expressed as flux control ratio, as percentage of the maximal respiratory capacity. E-F) Analysis of net and coupling ROUTINE achieved upon different experimental conditions and/or EVs, ACM or SNT treatment in differentiated SH-SY5Y. In this case, MPP^+ promoted a general and significative decrease of both net and coupling respirations.

However, no effect was observed upon EVs, ACM or SNT treatment. Data are expressed as a flux control ratio, as percentage of specific reference states maximal and basal respiratory capacity for net and coupling respiration, respectively. G-H) Analysis of net and coupling ROUTINE achieved upon different experimental conditions and/or EVs treatment in undifferentiated SH-SY5Y. As for differentiated cells, MPP^+ promoted a reduction of both parameters which is not restored by EVs. Data are expressed as a flux control ratio, as percentage of specific reference states maximal and basal respiratory capacity for net and coupling respiration, respectively. In (A-H) data are expressed as mean ± SD. One-way ANOVA with Tukey's multiple comparison was performed, with ∗p <0.05, **p<0.01 and ****p<0.001 (CTRL vs. MPP^+), ns: not significant.
